# The novel transcriptomic signature of angiogenesis predicts clinical outcome, tumor microenvironment and treatment response for prostate adenocarcinoma

**DOI:** 10.1186/s10020-022-00504-6

**Published:** 2022-07-14

**Authors:** Cheng-Yuan Gu, Bo Dai, Yao Zhu, Guo-Wen Lin, Hong-Kai Wang, Ding-Wei Ye, Xiao-Jian Qin

**Affiliations:** 1 Department of Urology, Fudan University Shanghai Cancer Center (FUSCC), Fudan University, No. 270 Dong’an Road, Shanghai, 200032 People’s Republic of China; 2 Department of Oncology, Shanghai Medical College, Fudan University, Shanghai, 200032 People’s Republic of China

**Keywords:** Prostate adenocarcinoma, Angiogenesis, TCGA, Transcriptomic signature, Survival analysis, Nomogram, Tumor microenvironment, Treatment response prediction, Targeted therapies

## Abstract

Angiogenesis plays the critical roles in promoting tumor progression, aggressiveness, and metastasis. Although few studies have revealed some angiogenesis-related genes (ARGs) could serve as prognosis-related biomarkers for the prostate cancer (PCa), the integrated role of ARGs has not been systematically studied. The RNA-sequencing data and clinical information of prostate adenocarcinoma (PRAD) were downloaded from The Cancer Genome Atlas (TCGA) as discovery dataset. Twenty-three ARGs in total were identified to be correlated with prognosis of PRAD by the univariate Cox regression analysis, and a 19-ARG signature was further developed with significant correlation with the disease-free survival (DFS) of PRAD by the least absolute shrinkage and selection operator (LASSO) Cox regression with tenfold cross-validation. The signature stratified PRAD patients into high- and low-ARGs signature score groups, and those with high ARGs signature score were associated with significantly poorer outcomes (median DFS: 62.71 months vs unreached, p < 0.0001). The predicting ability of ARGs signature was subsequently validated in two independent cohorts of GSE40272 & PRAD_MSKCC. Notably, the 19-ARG signature outperformed the typical clinical features or each involved ARG in predicting the DFS of PRAD. Furthermore, a prognostic nomogram was constructed with three independent prognostic factors, including the ARGs signature, T stage and Gleason score. The predicted results from the nomogram (C-index = 0.799, 95%CI = 0.744–0.854) matched well with the observed outcomes, which was verified by the calibration curves. The values of area under receiver operating characteristic curve (AUC) for DFS at 1-, 3-, 5-year for the nomogram were 0.82, 0.83, and 0.83, respectively, indicating the performance of nomogram model is of reasonably high accuracy and robustness. Moreover, functional enrichment analysis demonstrated the potential targets of E2F targets, G2M checkpoint pathways, and cell cycle pathways to suppress the PRAD progression. Of note, the high-risk PRAD patients were more sensitive to immune therapies, but Treg might hinder benefits from immunotherapies. Additionally, this established tool also could predict response to neoadjuvant androgen deprivation therapy (ADT) and some chemotherapy drugs, such as cisplatin, paclitaxel, and docetaxel, etc. The novel ARGs signature, with prognostic significance, can further promote the application of targeted therapies in different stratifications of PCa patients.

**Supplementary Information:**

The online version contains supplementary material available at 10.1186/s10020-022-00504-6.

## Introduction

Prostate adenocarcinoma (PRAD), accounting for 95% of all prostate cancers (PCa), is a complicated disease threatening the health of men worldwide (Rebello et al. 2021). As reported, PCa has become the second most common male malignancy, with a incidence of 29.3/10^5^ and morality of 7.6/10^5^ worldwide (Feng et al. 2019). Similarly, the incidence of PCa in China has been rising to over 10/10^5^ and now ranks the seventh most common male cancer and tenth leading cause of cancer death (Feng et al. 2019; Zhu and Ye 2020). With the development of screening techniques and modern medicine, prostate cancer has a favorable 5-year survival rate among the common types of cancers. However, patients with PCa in China are often diagnosed at locally advanced stage or metastasis stage, resulting in a high mortality-to-incidence ratio, nearly 50% (Zhu and Ye 2020). Though the 5 year-survival of patients with localized PCa is over 95%, about half patients who received radical prostatectomy will experience biochemical recurrences (Dell'Oglio et al. 1990). Thus, reliable prognostic biomarkers should be proposed to improve the clinical management of PCa.

Some risk stratification tools based on gene expression have been developed, such as Decipher (Spratt et al. 2017), Oncotype Dx Genomic Prostate Score (Eure et al. 2017) and Prolaris (Shore et al. 2016). These tools have been proved to be associated with disease recurrence after surgery and/or adjuvant therapy. However, some of these models did not make contributions to clinical practices (Cucchiara et al. 2018). To our knowledge, a number of molecular biomarkers have been discovered as well, which are capable of identifying aggressive subtypes of indolent PCa with higher specificity (Bertoli et al. 2016; Lam et al. 2020; Faisal and Lotan 2020; Zhuo et al. 2018). For instance, miRNA prognostic biomarkers, such as *let-7a*, *miR-141*, *miR-375* and *miR-182*, are correlated with lymph node metastasis and clinical staging in PCa (Bertoli et al. 2016). Moreover, epigenetic biomarkers, such as *GSTP1* and *APC*, were found to be correlated with the relapse of PCa (Lam et al. 2020). Next generation sequencing (NGS) technologies enabled us to have a deep understanding of the genomic information of PCa patients, and some genomic alteration signatures have been applied in diagnosis, prognosis, and clinical therapeutics (Faisal and Lotan 2020). Furthermore, increasing transcriptomic data of PCa patients provided deep insights into molecular mechanisms of tumorigenesis and progression, and also promoted the development of molecular biomarkers. For example, the upregulation of *SRPK2* was shown to affect cancer cell proliferation, migration, cell cycle, and was found to be correlated with a higher Gleason score, advanced clinical stage, tumor metastasis and poor prognosis (Zhuo et al. 2018). However, the prognosis and management of PCa are still complicated due to the molecular, cellular, and clinical heterogeneity in PCa (Chen et al. 2021). Therefore, more rigorous and reliable prognosis prediction models are urgently needed to stratify the PCa patients and improve the clinical outcomes.

Angiogenesis is well-known to play critical roles in the formation of nascent blood vessels, which are mainly involved in the vascular development and repair of damaged blood vessels (Rajabi and Mousa 2017). Whereas, pathological blood vessels, unlike normal blood vessels, are immature and can influence the tumor microenvironment (TME) and promote proliferation and migration of cancer cells and support tumor progression, invasion and distant metastasis (Katayama et al. 2019; Carmeliet and Jain 2000). Thus, pathological angiogenesis is considered as one of the tumor hallmarks, correlated with poor prognosis in many cancer types (Ramjiawan et al. 2017). Nowadays, plenty of ARGs had been identified to be involved in the development of tumors (Pavlakovic et al. 2001). The overexpression of some certain ARGs, such as vascular endothelial growth factor A (*VEGFA*), neuropilin-1 (*NRP1*) and PlexinA2 (*PLXNA2*), were found in PCa and their overexpression correlated with tumor metastasis and poorer prognosis (Melegh and Oltean 2019; Yin et al. 2021). In addition, some studies revealed that PCa progression and metastasis were promoted by other angiogenesis-enhancing factors (Zhao et al. 2021; Strohmeyer et al. 2000). These studies focused on the prognostic value of a single ARG or angiogenesis-related processes, but the roles of ARGs related to the whole angiogenesis activity in predicting the prognosis of PCa are not clearly clarified.

In our study, we investigated the RNA-seq and clinical features of PRAD samples downloaded from the TCGA, and 23 angiogenesis-related genes correlated with worse DFS were identified from the Hallmark Angiogenesis gene set (36 ARGs in total). LASSO Cox regression with tenfold cross-validation further confirmed a novel 19-ARG signature, which was a predominant prognostic factor for the DFS of PRAD. Furthermore, a nomogram based on the ARGs signature was generated to predict the prognosis of PRAD patients. Finally, the role of the signature in predicting treatment response was investigated to explore the potential targeted therapies for PRAD patients.

## Methods and materials

### Data resources and selection of angiogenesis-related genes

The original genomic alternations, mRNA expression and clinical features of 501 PRAD samples (4/501 PRAD samples lacking one of these information were excluded, only 497/501 PRAD samples were included in the following analyses) in PRAD cohort (TCGA, Firehose Legacy) were downloaded via the cbioportal (https://www.cbioportal.org/study/summary?id=prad_tcga) as the discovery dataset (Table 1). GSE40272 dataset, which contains 89 PRAD samples, was downloaded from the GEO (https://www.ncbi.nlm.nih.gov/geo/query/acc.cgi?acc=GSE40272) as the first validation dataset (Additional file 7: Table S1), meanwhile, the PRAD_MSKCC cohort, including a total of 156 PRAD samples with mRNA expression data and there were 140/156 PRAD samples with DFS information), retrieved via the cbioportal was regarded as another validation dataset (Additional file 8: Table S2).

**Table 1:** Clinical features of 497 PRAD patient samples from the TCGA

Characteristics		Number (%)
Total		497
Age	Median (range)	61 [41, 78]
PSA	Median (range)	0.1 [0, 323]
Gleason score	Primary + secondary	
	6	45
	7	247
	8	64
	9	137
	10	4
T stage	T1	177
	T2	173
	T3	53
	T4	2
	NA	92
N stage	N0	345
	N1	79
	NA	73
M stage	M0	455
	M1	3
	NA	39

*PRAD* prostate adenocarcinoma, *NA* not applicable

The Angiogenesis-related gene set, including 36 ARGs in total, were downloaded from Molecular Signatures Database (http://www.gsea-msigdb.org/gsea/msigdb/, MSigDB-Hallmark version 7.4).

### Differential expression analysis between PRAD patients and normal people, and expression and mutation profiles of ARGs in PRAD

The mRNA expression data in the cohort (containing 500 tumor samples & 51 normal samples), namely GDC TCGA Prostate Cancer (PRAD) that was collected in the website of UCSC xena (https://xenabrowser.net/datapages/) and could be directly downloaded from the following: https://gdc-hub.s3.us-east-1.amazonaws.com/download/TCGA-PRAD.htseq_counts.tsv.gz, were retrieved to explore the differentially expressed ARGs using the “DeSeq2” package, while |log_2_ (Fold Change) |> 1 and p < 0.05 were considered significantly differentially expressed.

Angiogenesis-related genes data were extracted using cBioPortal to show the relationship between genetic alterations (missense, splice, truncating, amplification, and deep deletion alterations, etc.) and patients’ survival or between expression alterations and patients’ survival among these patients from the TCGA-PRAD cohort. The relatively high or low expression was defined by the custom parameter (z-score) of cBioPortal, while “z-score > 2” represented the relatively high mRNA expression and “z-score < − 2” represented the relatively low mRNA expression.

### Construction of the angiogenesis-related prognostic signature and nomogram

The ARGs related to DFS (P < 0.05) were identified via a univariate Cox regression analysis using optimized algorithm. Following least absolute shrinkage and selection operator (LASSO) Cox regression with tenfold cross-validation was performed using the “glmnet” package in R studio (Sauerbrei et al. 2007; Wang et al. 2019). Multivariate Cox regression analysis was then conducted for the construction of this ARG signature, and the ARGs signature score formula was established. ARGs signature score = Expression level of gene 1 × ɑ_1_ + Expression level of gene 2 × ɑ_2_ + Expression level of gene 3 × ɑ_3_ + … + Expression level of gene n × ɑ_n_, where ɑ_n_ denotes the coefficient for the corresponding gene in this model. The cutoff value was drawn from median ARGs signature score to sort the patient samples into high- and low-ARGs signature score groups. The survival curves were drawn using the “survival” package in R studio. The analysis of receiver operating characteristic curve (ROC) was demonstrated by the “timeROC” package in R studio, and the values of area under ROC curve (AUC) for DFS were used to evaluate the ability of ARGs signature or clinical feature in prognosis prediction.

Underlying ARGs signature, clinical association analysis was subsequently conducted. Moreover, ARGs signature and clinical features, such as diagnosis age, PSA (prostate specific antigen) level, Gleason score and T, N, M stage, were further enrolled in the multivariate Cox regression analysis to determine the independent prognostic factors. Based on the independent prognostic factors, the nomogram was constructed via the Cox regression model by using the “rms” package in R studio (Chun et al. 2006). Also, the calibration plots were drawn by the “rms” package in R studio to estimate the accuracy of predicted DFS by the nomogram.

### Genomic alteration and functional enrichment analysis

Then, the genomic alteration profiling between high- and low-ARGs signature score groups was compared. Initially, the comparison analysis of mutation count between these two groups was conducted by using the “violinplot” package in R studio. Subsequently, the oncoplots were drawn by using the “maftools” package in R studio to exhibit genomic alteration landscapes of high- and low-ARGs signature score groups. The oncogenic genes involved in the DDR, PI3K, and Wnt signaling pathways were highly correlated with the development and progression of PCa (Armenia et al. 2018), thus, genomic alterations in these three pathways were further investigated in the present study.

To explore the biological processes involved in the high- and low-ARGs signature score groups, we used the “clusterProfiler” package (Yu et al. 2012) in R studio to perform Hallmark gene set enrichment analysis (http://www.gsea-msigdb.org/gsea/msigdb/) and Kyoto Encyclopedia of Genes and Genomes (KEGG, https://www.kegg.jp/) pathway enrichment analysis, and all of these analyses were visualized by “ggplot2” and “enrichplot” in R studio. The threshold was set at an adjusted p-value < 0.05.

### Evaluation of TME between high- and low-ARGs signature score groups

Transcriptomic data from the TCGA-PRAD cohort were used to compare the proportions of infiltration among 22 types of tumor-infiltrating immune cell by a deconvolution algorithm (CIBERSORT: Cell type Identification By Estimating Relative Subsets Of known RNA Transcripts) between the high- and low-ARGs signature score groups. Furthermore, immune and stromal scores illustrating the infiltration status of TME cells were calculated using ESTIMATE (Estimation of STromal and Immune cells in MAlignant Tumours using Expression data) (Yoshihara et al. 2013a). In addition, the expression levels of immune checkpoint genes, including *PD-L1*, *PD-1*, *CTLA4*, and *TIM3*, were compared between the high- and low-ARGs signature score groups. Moreover, the relationship between the expression levels of immune checkpoint genes and ARGs signature score (or signature-related ARGs) was further investigated by the correlation analysis.

### The analyses of endocrine therapy and chemotherapy responses

The GSE102124 dataset containing 19 primary prostate cancer samples with the treatment of neoadjuvant ADT and 3 primary prostate cancer control samples (Sowalsky et al. 2018) and the GSE74685 dataset containing 117 castration-resistant prostate cancer (CRPC) samples with the treatment of luteinizing hormone-releasing hormone (LHRH) agonist, 19 CRPC samples receiving the orchiectomy treatment, and 13 CRPC control samples (Kumar et al. 2016) were retrieved from the GEO for the analysis of the ADT treatment responses.

Meanwhile, data were retrieved from the Genomics of Drug Sensitivity in Cancer (GDSC, https://www.cancerrxgene.org/) to determine the difference in the response to chemotherapy between high or low-ARGs signature score groups (Yang et al. 2013a; Iorio et al. 2016; Garnett et al. 2012). In the present study, the half-maximal inhibitory concentration (IC50) was utilized to evaluate the drug response. Meanwhile, the IC50 of each sample was estimated between the high- and low-ARGs signature score groups. Furthermore, mRNA expression data of docetaxel sensitive and resistant variants of PC3 PCa cell line from GSE140440 (Schnepp et al. 2020) was further employed to testify the predictive role of ARGs in chemotherapy response.

### Immunohistochemistry (IHC) analysis

The protein expression of 19 ARGs involved in the signature, among TCGA-PRAD samples, was analyzed by IHC using the available scanned tumor staining from Human Protein Atlas (HPA) database (https://www.proteinatlas.org/). The information of IHC staining was determined and manually adjusted by experts from the HPA database, and ARGs IHC staining was defined and exhibited as high, medium, low staining or not detected. A total of 9–12 PRAD samples were enrolled in the analysis of ARGs IHC staining. In addition, there existed the IHC staining of 16 ARGs protein expression, except CXCL6, OLR1, and LPL, retrieved from the HPA database.

### Statistical analyses

**Fig. 1:**
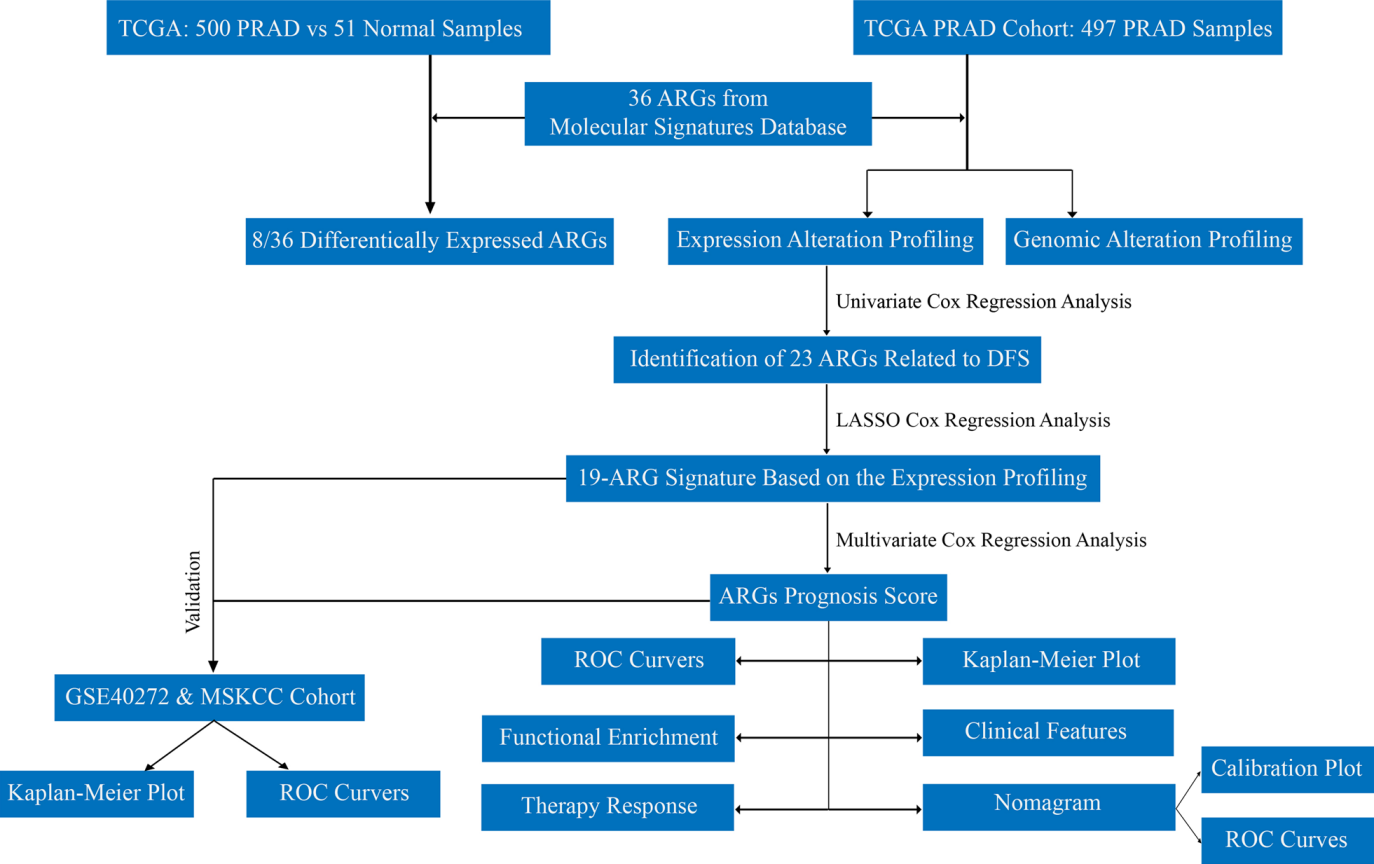
A flow chart showing a schematic representation for the construction of a novel angiogenesis-related 19-gene signature model

Chi-square, Fisher test, and Wilcoxon rank test statistical analyses were performed using R software (v. 3.4.3, https://rstudio.com/). A (adjust) p-value < 0.05 was considered statistically significant. A log-rank test was used to estimate the survival curves between the high- and low-ARGs signature score groups. A schematic representation for the design of the present study was shown in Fig. 1.

## Results

### Genomic and expression alterations of ARGs in PRAD patients

**Fig. 2:**
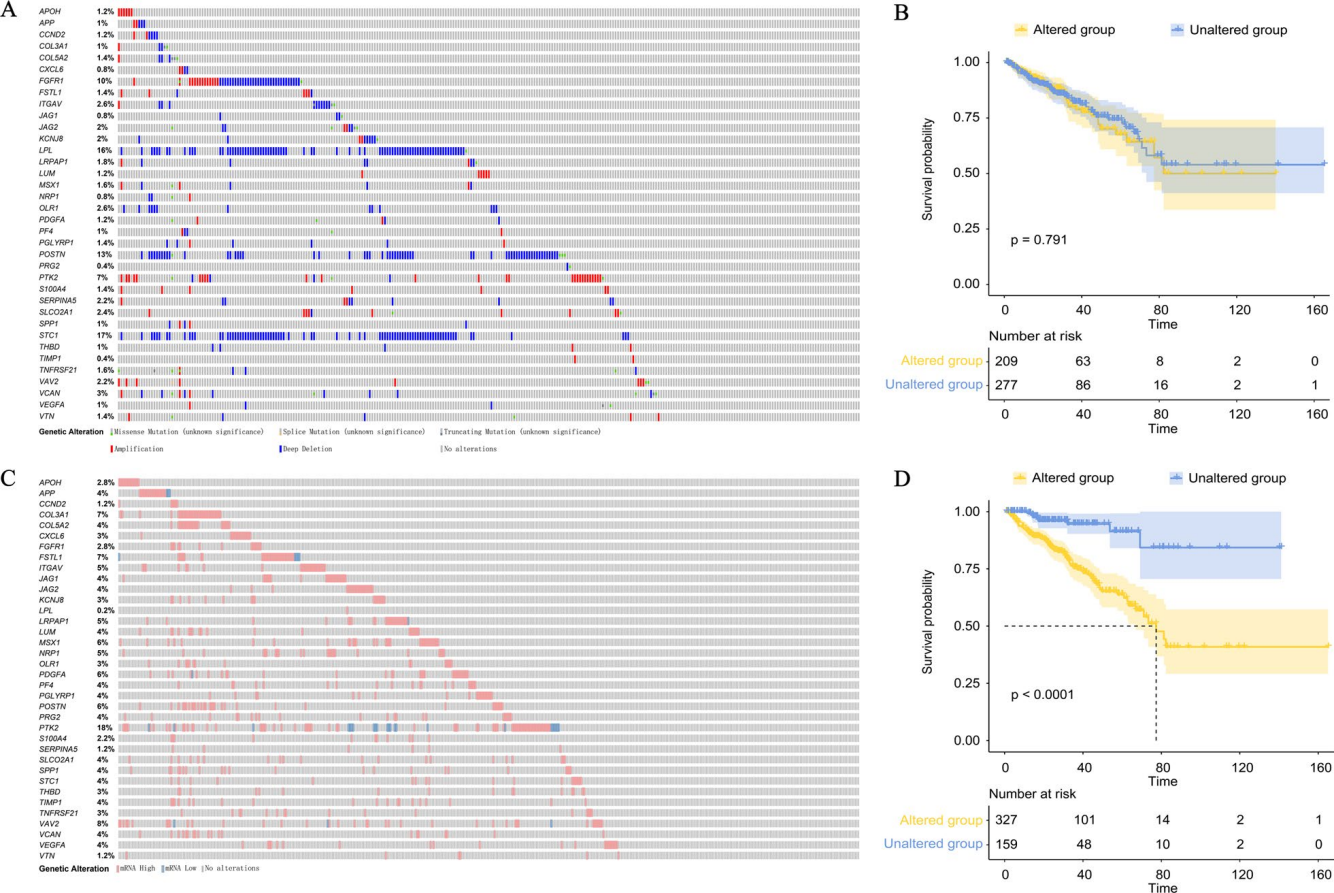
Exploration of the ARGs mutation and expression profiles in PRAD patients from the TCGA database. **A** cBioPortal oncoprint of ARGs mutation profiling in each PRAD patient. **B** Kaplan–Meier DFS curves of groups (log-rank test) with (N = 209) or without (N = 277) altered mutations. **C** cBioPortal oncoprint of ARGs expression profiling in each PRAD patient. **D** Kaplan–Meier DFS curves of groups (log-rank test) with altered expression of ARGs (N = 327) or not (N = 159)

Initially, we analyzed the genomic alteration profiling of ARGs gene set in PRAD patient samples from the TCGA database, and it was showed that 43% (214/497) of PRAD patient samples had at least one genomic alteration, including missense, splice, truncating, amplification, and/or deep deletion alterations in ARGs (Fig. 2A). Among those altered genes, *STC1* was the most prevalent (17%), while others were altered in 0.4%-16% of patient samples. However, there was no significant difference (p = 0.791) in the DFS between groups with or without genetic mutations in ARGs (Fig. 2B).

Then, we explored the expression data of the ARGs in PRAD patient samples from TCGA. Almost 68% (336/497) of PRAD patient samples had altered expression of ARGs (Fig. 2C). Notably, patient samples with the altered expression of ARGs had significantly poorer DFS than that of patient samples without changes (median DFS: 77.33 months vs unreached, p < 0.0001, Fig. 2D). While the comparison of expression data between tumor and normal samples showed that 8 out of 36 ARGs were significantly differentially expressed, including *CCND2*, *CXCL6*, *KCNJ8*, *LPL*, *SERPINA5*, *SLCO2A1*, *VTN,* and *APOH* (Additional file 9: Table S3). Interestingly, only *APOH* was downregulated but other 7 ARGs were upregulated in the tumor samples, compared to the normal samples.

### Construction and evaluation of a novel prognostic signature for PRAD patients based on the ARGs expression profiling

**Fig. 3:**
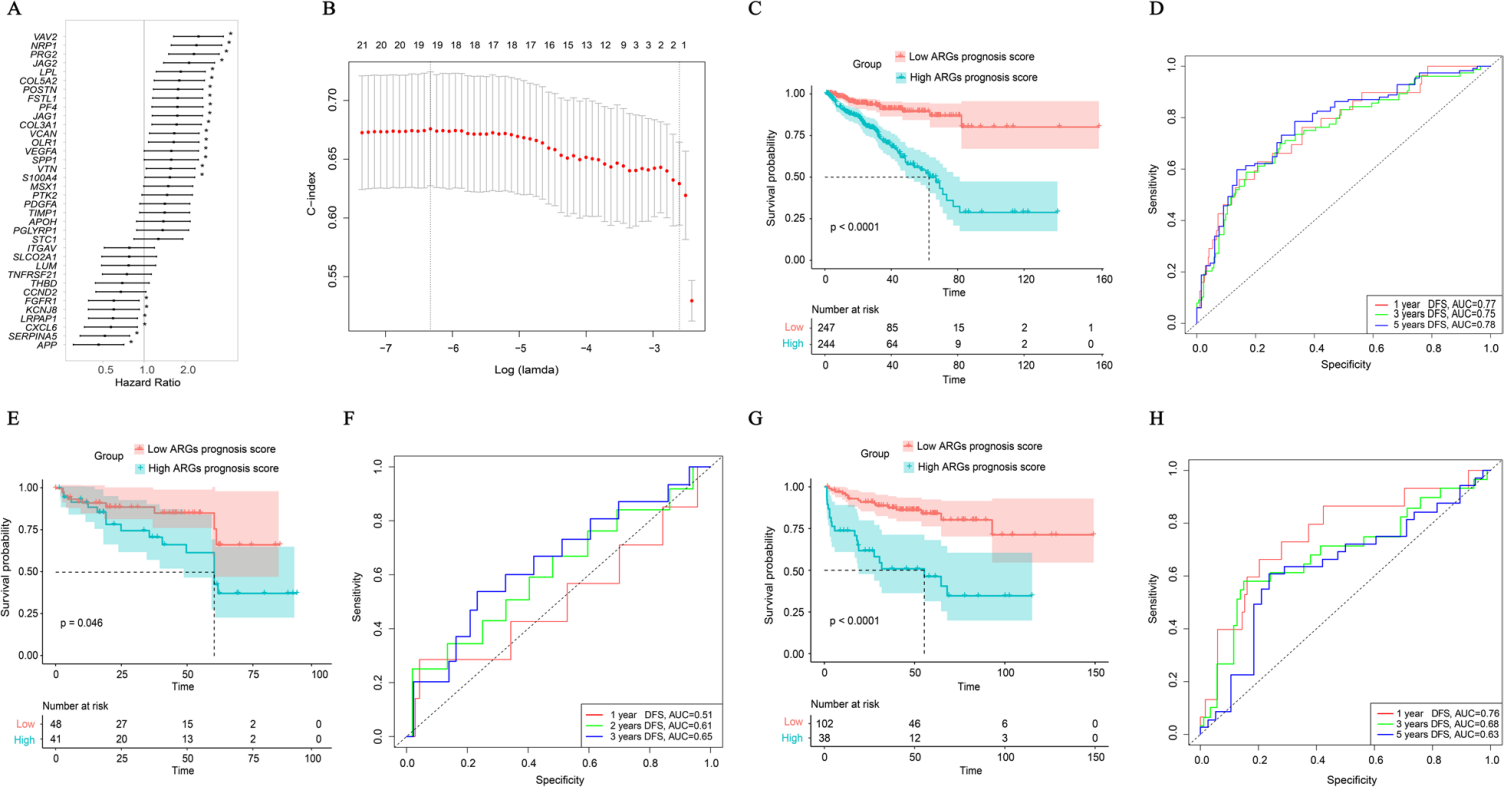
Construction and evaluation of a prognostic 19-ARG signature. **A** Hazard ratio in high and low expressions of ARGs in PRAD cohort, and corresponding 95% confidence intervals were calculated by the univariate regression analysis. **B** A 19-ARG signature was confirmed by the LASSO regression analysis with tenfold cross-validation. **C** Kaplan–Meier DFS curves for patients from the TCGA cohort in the high- or low-ARGs signature score group. **D** The ROC curves for DFS at 1, 3, and 5 years were analyzed in the TCGA cohort. **E** Kaplan–Meier DFS curves for patient samples assigned to high- and low-ARGs signature score in the validation group, GSE40272 from the GEO database. **F** The ROC curves for DFS at 1, 2, and 3 years were analyzed in GSE40272. **G** Kaplan–Meier DFS curves for patient samples assigned to high- and low-ARGs signature score in another validation group of the PRAD_MSKCC cohort. **H** The ROC curves for DFS at 1, 3, and 5 years were analyzed in the PRAD_MSKCC cohort

The univariate Cox regression analysis was used to identify the specific association between each individual ARG gene from the ARGs gene set and the DFS of PRAD patient samples from the TCGA cohort, and we found a total of 23 ARGs were significantly correlated with the DFS of PRAD patients (p < 0.05, Fig. 3A). Among these identified ARGs, the overexpression of 17 ARGs were associated with poorer DFS of PRAD patients; on the contrary, the expression of 6 ARGs were related to the prolonged DFS in the PRAD patients.

By the LASSO Cox regression analysis, 19 ARGs were further determined to construct a novel prognostic signature for PRAD patients (Fig. 3B). The coefficients of these 19 ARGs were calculated via the multivariate Cox regression analysis (Table 2), and a median ARGs signature score was defined as a cutoff value which was used to divide PRAD patient samples into the high- and low-ARGs signature score groups. Notably, patients in the high ARGs signature score group had the significantly worse prognosis (median DFS: 62.71 months vs unreached, p < 0.0001, Fig. 3C), compared to those in the low ARGs signature score group. Moreover, the AUC values for DFS at 1, 3, and 5 years were 0.77, 0.75 and 0.78, respectively (Fig. 3D).

**Table 2:** Hazard ratios and coefficients of DFS-related ARGs

Gene	Hazard ratio	95%CI	Coefficient	p-value
*VAV2*	2.50	1.64–3.80	3.3924	< 0.05
*NRP1*	2.41	1.58–3.70	0.5622	
*PRG2*	2.31	1.51–3.54	1.1902	
*JAG2*	2.13	1.39–3.27	0.3202	
*LPL*	1.85	1.23–2.80	- 0.0801	
*POSTN*	1.77	1.17–2.69	0.3631	
*FSTL1*	1.76	1.17–2.69	0.7902	
*PF4*	1.75	1.14–2.69	0.6997	
*JAG1*	1.75	1.15–2.67	0.2249	
*COL3A1*	1.73	1.14–2.62	0.3681	
*OLR1*	1.65	1.08–2.52	− 0.0198	
*VEGFA*	1.58	1.01–2.49	0.1602	
*VTN*	1.56	1.03–2.36	0.0476	
*S100A4*	1.96	1.17–3.29	0.9752	
*FGFR1*	0.60	0.39–0.92	− 1.1699	
*LRPAP1*	0.59	0.39–0.89	1.1291	
*CXCL6*	0.57	0.37–0.89	− 0.1911	
*SERPINA5*	0.52	0.34–0.78	− 0.4216	
*APP*	0.46	0.30–0.71	− 0.3995	

*DFS* disease-free survival, *CI* confidence interval, *NA* not applicable

In addition, the constructed signature was further verified in two independent validation cohorts: GSE40272 dataset & PRAD_MSKCC cohort. The Kaplan–Meier curve for PRAD patients in GSE40272 showed that patients with high ARGs signature score had the worse survival (median DFS: 60.30 months vs unreached, p < 0.05, Fig. 3E), in contrast to those with low ARGs signature score. Besides, the AUC values for DFS at 1, 2, and 3 years were 0.51, 0.61 and 0.65, respectively (Fig. 3F). In addition, the survival analysis of PRAD patients from PRAD_MSKCC cohort also demonstrated that patients in high ARGs signature score group had the poorer outcomes (median DFS: 55.39 months vs unreached, p < 0.0001, Fig. 3G), and the AUC values for DFS at 1, 3, and 5 years were 0.76, 0.68 and 0.63, respectively (Fig. 3H).

### Association analysis between the ARGs signature and clinical features

**Fig. 4:**
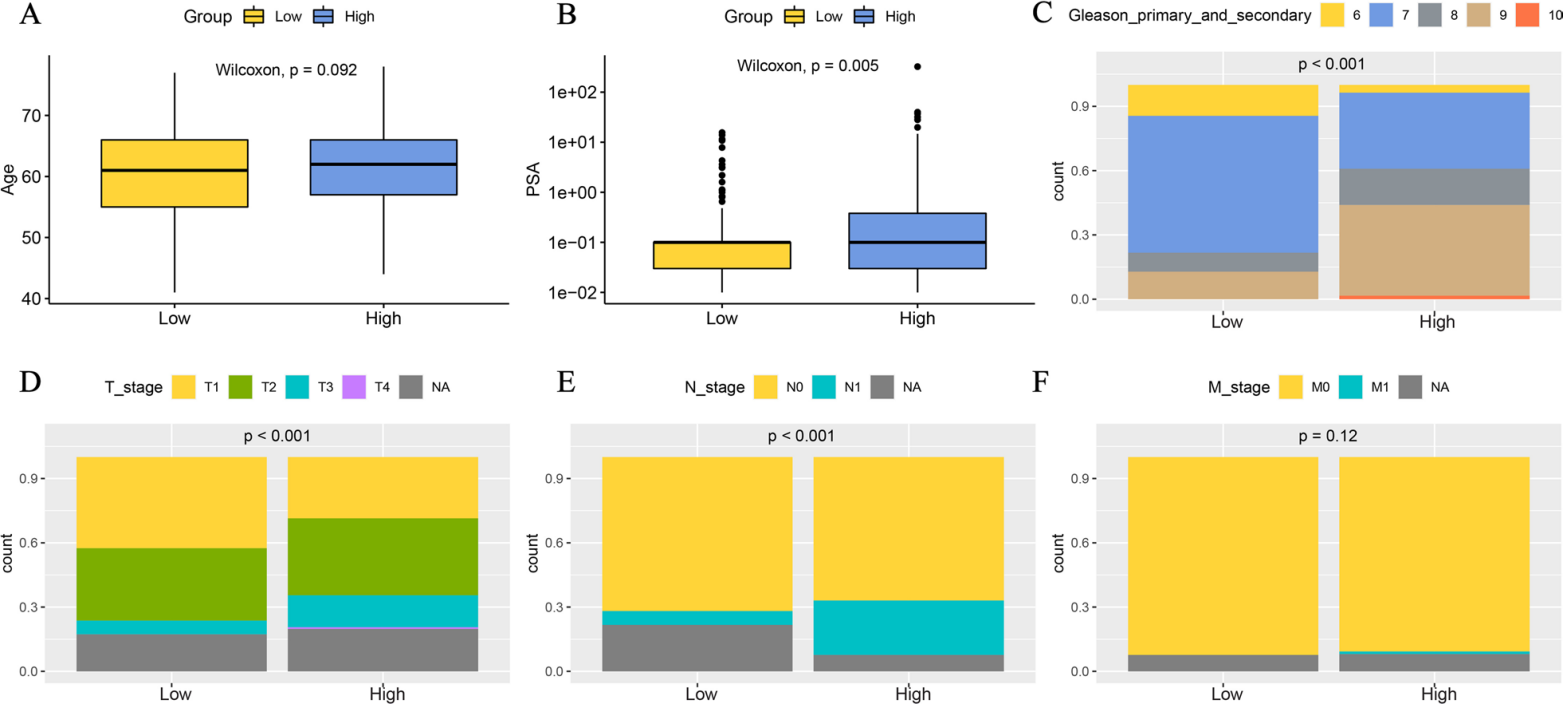
The analyses of clinical features between high- and low-ARGs signature score groups in PRAD cohort. **A** Boxplots representing age of diagnosis for high- and low-ARGs signature score groups (p = 0.092). **B** Boxplots of the PSA level between high- and low ARGs signature score groups (p = 0.005). **C** Percentage-staked bar plot representing patient distribution with Gleason primary + secondary score between high- and low-ARGs signature score groups (p < 0.001). **D** Percentage-staked bar plot representing tumor stage (T1/T2/T3/T4/NA) between high- and low-ARGs signature score groups (p < 0.001). **E** Percentage-staked bar plot representing lymph node status (N0/N1//NA) (p < 0.001) between high- and low-ARGs signature score groups (p < 0.001). **F** Percentage-staked bar plot representing tumor metastasis (M0/M1//NA) between high- and low-ARGs signature score groups (p = 0.12). (*NA* not applicable)

Next, we investigated the clinical features related to ARGs signature in the TCGA-PRAD cohort. There was no significant difference in the age at diagnosis between two groups (median age at diagnosis: 61.02 vs 60.75 years old, p > 0.05, Fig. 4A), but the PSA level (PSA level: 0.1 ng/mL [0.03, 0.365] vs 0.1 ng/mL [0.03, 0.10], p = 0.005) was significantly higher in the patients with high ARGs signature score (Fig. 4B).

Gleason score is an extensively used marker for histological grading in PCa, thus, we investigated the difference in the distribution of Gleason score between patients with different ARGs signature score. It was found that more PRAD patients in the high ARGs signature score group had a high Gleason score (16.94% vs 8.87% in Gleason 8, 42.34% vs 12.91% in Gleason 9, 1.61% vs 0% in Gleason 10), compared to those in low ARGs signature score group (p < 0.01, Fig. 4C). Further comprehensive analyses of clinical stages revealed that patients with high ARGs signature score were significantly associated with advanced tumor stages and lymph node invasion. Among these patients with high ARGs signature score, 14.92% and 25.41% of them had disease of T3 and N1, respectively (p < 0.01, Fig. 4D, E). In contrast, only 6.4% of patients with low ARGs signature score were at T3 stage and/or N1 stage. No significant difference in distant metastasis between two groups was observed due to the limited patients in M1 stage involved in the TCGA dataset (Fig. 4F). Of note, it was observed that the expression of common tumor angiogenesis markers of *CD34* & *CD105* was higher in high ARGs signature score group, moreover, ARGs signature score was positively with the expression of *CD34* & *CD105* (p < 0.001, Additional file 1: Fig. S1).

### Construction of a nomogram

**Fig. 5:**
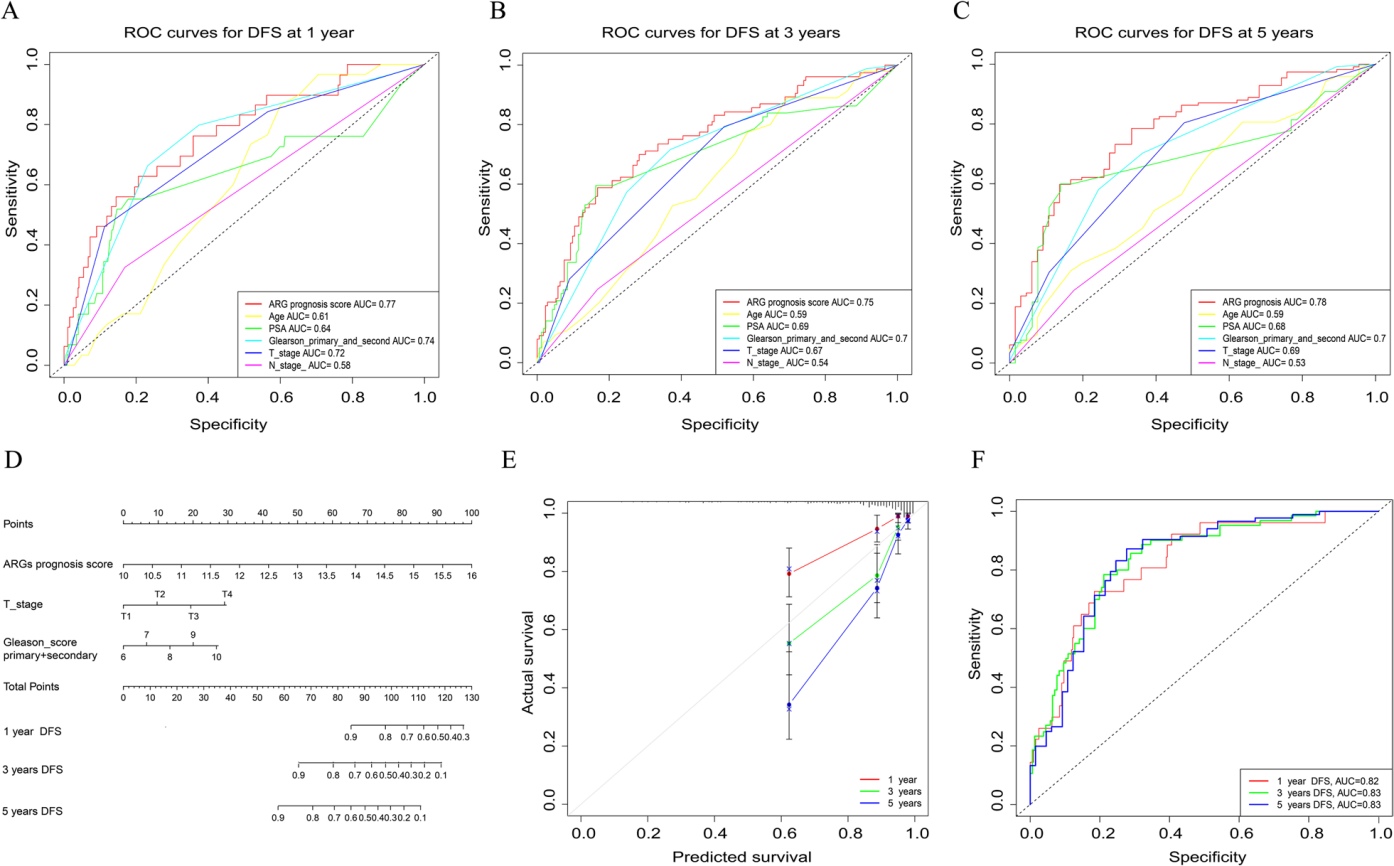
Construction and evaluation of a nomogram. ROC curves, for DFS at **A** 1 year, **B** 3 years, **C** 5 years, represent the sensitivity and specificity of a 19-ARG signature and the selected clinical features in predicting the prognosis of PRAD patients. **D** A nomogram for predicting DFS possibilities of PRAD patients. **E** Calibration plots revealed the agreement between the predicted outcomes and actual disease-free survival at 1, 3, and 5 years in the TCGA-PRAD cohort. **F** ROC curves, for DFS at 1, 3, and 5 years, represent the sensitivity and specificity of a nomogram

Compared to the representative clinical features, the ARGs signature score, Gleason score, and T stage were shown to be the predominant prognostic factors for PRAD patients by the multivariate Cox regression analysis and the ROC curve analysis (Table 3). The AUC values for DFS at 1, 3, and 5 years showed that the specificity and sensitivity of the 19-ARG signature were higher than those of clinical features (Fig. 5A–C). Besides, the ARGs signature outperformed a single ARG involved in this signature to predict the 1, 3, and 5 years DFS of PRAD patients (Additional file 2: Fig. S2). Subsequently, a nomogram based on the ARGs signature score was established, together with the clinical parameters: T stage and “Gleason primary score + secondary score” (Fig. 5D). While the concordance index (C-index) of the nomogram was 0.799 (95%CI = 0.744–0.854). As indicated in the nomogram, every PRAD patient would have a total score and the patients with higher total score could have worse prognosis. In addition, the calibration plots revealed that the predicted outcomes were basically in agreement with the observed DFS in the TCGA-PRAD cohort (Fig. 5E). Meanwhile, the AUC values of 1-, 3-, 5-year DFS for the nomogram were 0.82, 0.83, and 0.83, respectively, showing that the nomogram had better and more stable predicting ability (Fig. 5F). Of note, the AUC values of 1-, 2-, 3-year DFS for the nomogram in GSE40272 were 0.59, 0.72, and 0.76, respectively, furthermore, the AUC values of 1-, 3-, 5-year DFS for the nomogram in the PRAD_MSKCC cohort also increased to 0.79, 0.74, and 0.68, by comparison with the performance of ARGs signature in prognosis prediction (Additional file 3: Fig. S3).

**Table 3:** The multivariate Cox regression analysis for the ARGs signature and clinical features

Index	Hazar ratio	95%CI	p-value
ARGs signature score	2.88	2.07–4.00	< 0.001
Age	1.00	0.97–1.00	0.820
PSA	1.02	0.98–1.10	0.335
Gleason score primary + secondary	1.54	1.14–2.10	0.005
T stage	1.78	1.27–2.50	0.001
N stage	0.73	0.41–1.30	0.293

*CI* confidence interval

### Enrichment of genomic alterations

**Fig. 6:**
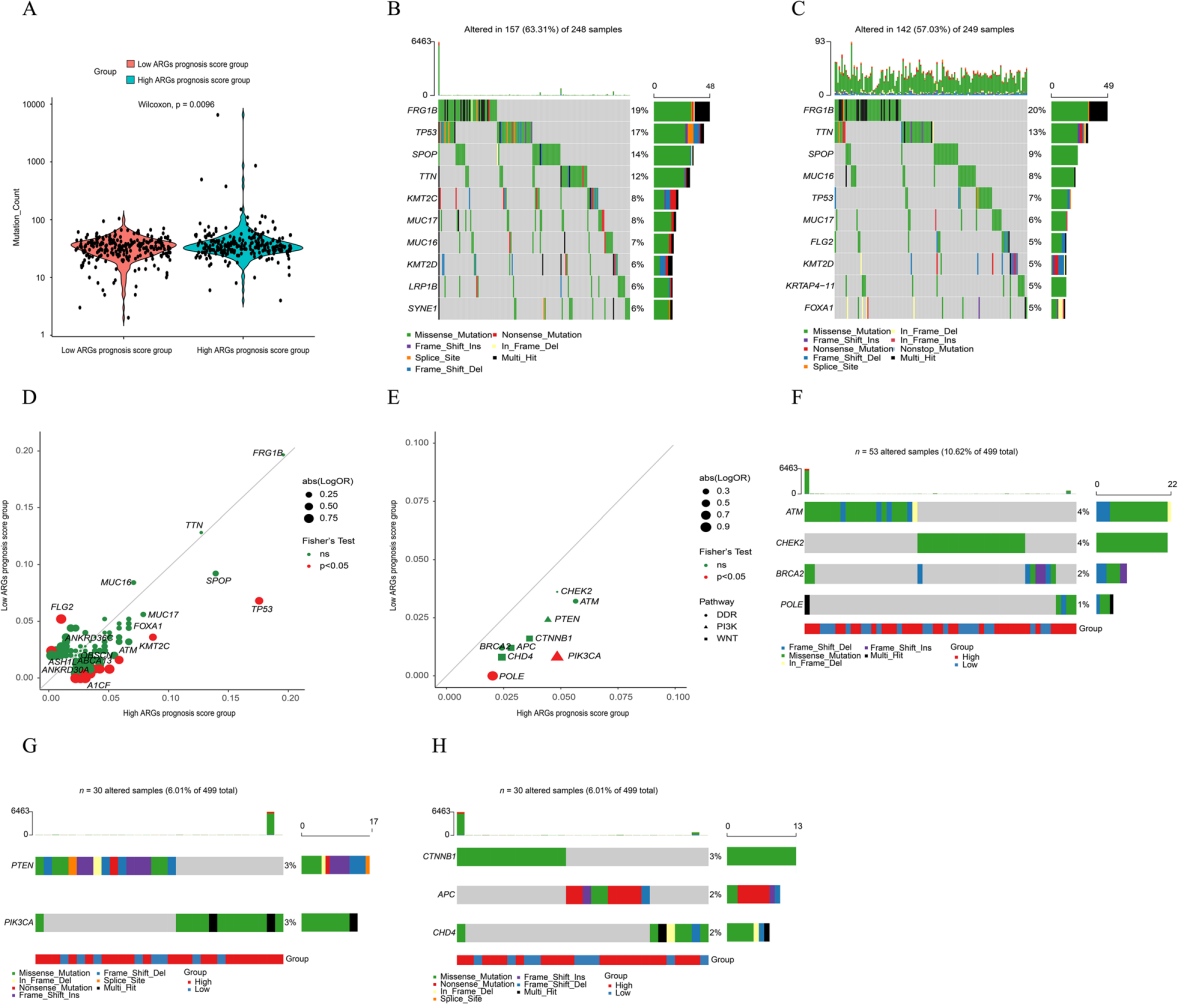
Genomic alteration enrichment analysis. **A** Vioplots of gene mutation counts in high- and low-ARGs signature score groups. **B** The alteration profile of the top 10 most frequently altered genes in high-ARGs signature score group. **C** The alteration profile of the top 10 most frequently altered genes in low-ARGs signature score group. **D** The distribution of the altered genes between the high- and low-ARGs signature score groups. **E** The distribution of the altered signaling pathways between the high- and low-ARGs signature score groups. **F** The alteration profile of the frequently altered genes of DDR, PI3K, and Wnt pathways between the high- and low-ARGs signature score groups

As is shown in Fig. 6A, more genomic mutations (mutation count: 35 [4, 6524] vs 34 [2, 104], p < 0.01) were presented in patients from high ARGs signature score group. Then we deeply investigated the mutation profiles of the top ten most frequently altered genes in high- and low-ARGs signature score groups. Obviously, the most prevalently altered genes in high ARGs signature score group were significantly different from those in the low ARGs signature score group (p < 0.05, Fig. 6B, C, Additional file 10: Table S4). Consequently, the systematic investigation was further conducted to explore which events were correlated with the high-risk PRAD patients. Genomic alterations happened more frequently in the high ARGs signature score group, and there was a statistically significant difference of genomic alterations in a total of 47 altered genes between the high- and low-ARGs signature score groups (Additional file 11: Table S5). By statistical analysis, 44 of 47 altered genes, including *TP53*, *PIK3CA* and *ALMS1*, etc., were significantly enriched in the high ARGs signature score group (p < 0.05, Fig. 6D, Additional file 11: Table S5), reversely, the low ARGs signature score group had a significant enrichment of only 3 altered genes, including *FLG2*, *KHDC4* (*KIAA0907*), and *KRTAP4-6* (p < 0.05, Fig. 6D, Additional file 11: Table S5). For the level of signaling pathway, we found that more frequently altered pathways of DNA Damage Response (DDR), PI3K and Wnt were enriched in the high ARGs signature score group, meanwhile, genes *PIK3CA* in PI3K signaling pathway and *POLE* in DDR signaling pathway were more frequently altered in the high ARGs signature score group (p < 0.05, Fig. 6E), and the altered genes were excluded as of the frequency < 1%. In addition, the DDR, PI3K and Wnt pathways were altered in nearly 10.62%, 6.01%, and 6.01% of the PRAD patient samples from the TCGA-PRAD cohort (Fig. 6F–H).

### Functional enrichment analysis

**Fig. 7:**
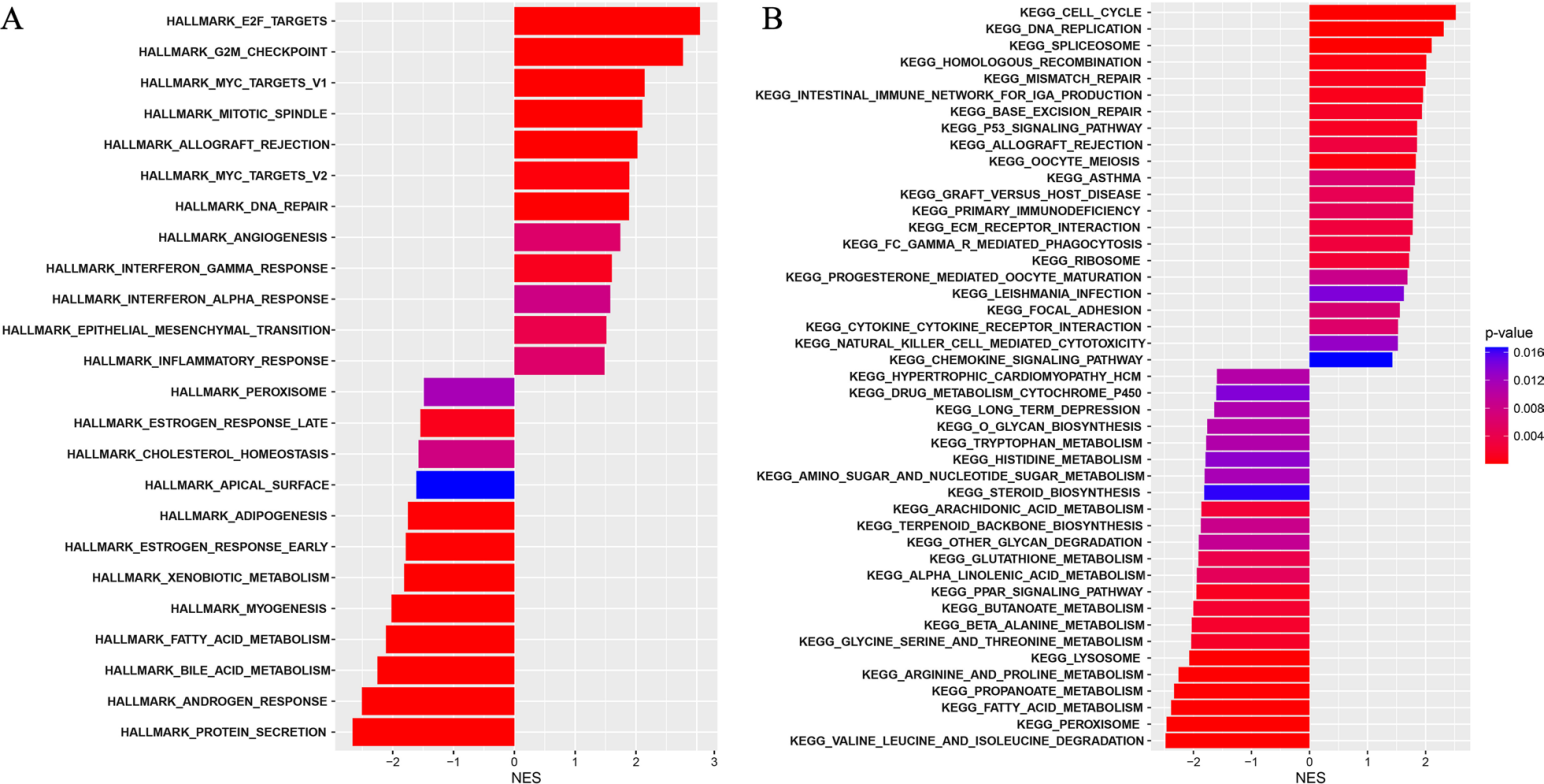
**A** Hallmark gene set enrichment analysis and **B** KEGG pathway enrichment analysis between high- and low-ARGs signature score groups in PRAD cohort

Initially, Hallmark gene set (Additional file 12: Table S6) and KEGG (Additional file 13: Table S7) enrichment analysis underlying the expression of signature-related ARGs revealed that the aberrant regulation of ARGs expression in PRAD samples was highly correlated with biological activities of E2F targets, G2M checkpoint, Myc targets V1, oxidative phosphorylation, cell cycle, angiogenesis, Androgen or estrogene response, extracellular matrix, and immune-related signaling pathways, etc. Based on the integrative ARGs signature, the Hallmark gene set enrichment analysis showed that E2F targets, G2M checkpoint, Myc targets V1, mitotic spindle, allograft rejection, DNA repair, Myc targets V2, interferon γ response, angiogenesis, epithelial-mesenchymal transition, inflammatory response, and interferon α response were highly enriched in the high ARGs signature score group (adjusted p-value < 0.01, Fig. 7A; Additional file 14: Table S8). And KEGG pathway enrichment analysis indicated that pathways related to cell cycle, DNA replication, and spliceosome etc., were abundantly enriched in the high ARGs signature score group (adjusted p-value < 0.01, Fig. 7B; Additional file 15: Table S9).

### The estimate of tumor microenvironment and ARGs signature-related immune checkpoint gene expression analysis

**Fig. 8:**
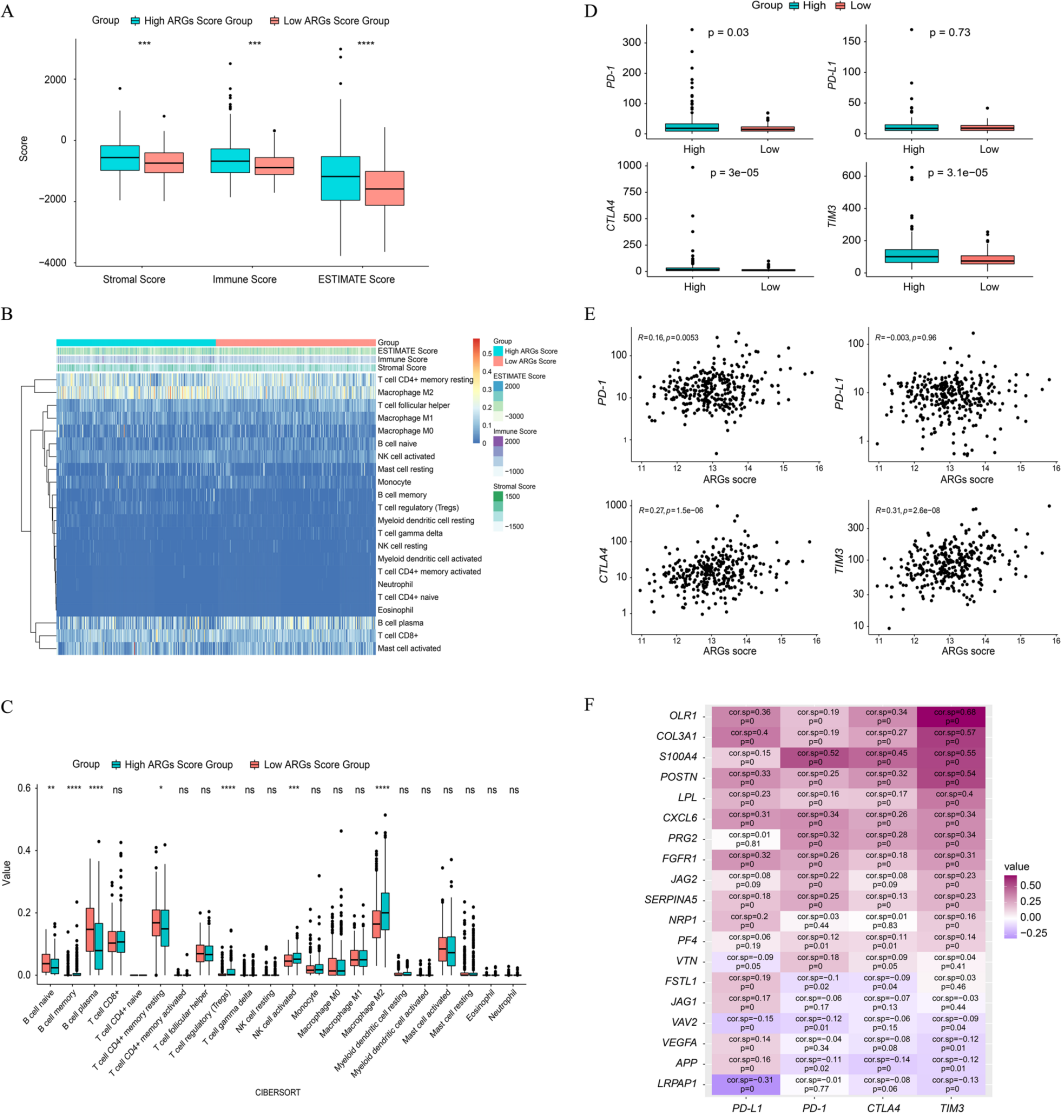
Tumor microenvironment (TME) in high- and low-ARGs signature score groups in PRAD cohort. **A** Comparison of the immune score, stromal score, and ESTIMATE score between the high- and low-ARGs signature score groups. **B** The immune profiles of high- and low-ARGs signature score groups. **C** Comparison of infiltrating immune cells between high- and low-ARGs signature score groups. **D** Differential expression of immune checkpoint genes (*PD-L1*, *PD-1*, *CTLA4*, *TIM3*) between high- and low-ARGs signature score groups. **E** Correlation analysis between the expression level of immune checkpoint genes (*PD-L1*, *PD-1*, *CTLA4*, *TIM3*) and the ARGs signature score. **F** Correlation analysis between the signature-related ARG and immune checkpoint genes. (*p < 0.05, **p < 0.01, ***p < 0.001, ****p < 0.0001, cor.sp represented “Spearman Correlation Coefficient”)

The stromal score, immune score and ESTIMATE score were significantly higher in high ARGs signature score group (p < 0.05, Fig. 8A). Meanwhile, infiltrating immune cells were analyzed to describe the immune profiles in the high- and low-ARGs signature score groups (Fig. 8B, C). Naive B cells, plasma B cells and CD4 + memory resting T cells are significantly enriched in the low ARGs signature score group. On the contrary, the high ARGs signature score group had a significantly higher abundance of memory B cells, Treg cells, activated NK cells, and Macrophage M2 (p < 0.05, Fig. 8C). Additionally, the expression of immune checkpoint genes was investigated, and which demonstrated that the expression of *PD-1*, *CTLA4*, and *TIM3* was significantly higher in the high ARGs signature score group (p < 0.05, Fig. 8D), however, the expression of *PD-L1* was nearly equivalent between two groups (p = 0.73, Fig. 8D) and was not associated with ARGs signature score (p = 0.96, Fig. 8E). Of note, it was found that the ARGs signature score had the extremely positive correlation with the expression of *PD-1* (p < 0.01), *CTLA4* (p < 0.0001), and *TIM3* (p < 0.0001) (Fig. 8E). Correlation analysis between each signature-related ARG and the selected immune checkpoint genes further demonstrated that *OLR1*, *COL3A1*, *S100A4*, *POSTN*, *LPL*, *CXCL6*, *FGFR1*, *JAG2*, and *SERPINA5* were all positively correlated with *PD-1*, *CTLA4*, and *TIM3* (p < 0.05, Fig. 8F), while *APP* was negatively correlated with *CTLA4* and *TIM3* but positively correlated with *PD-1* (p < 0.05, Fig. 8F).

### The evaluation of endocrine therapy and chemotherapy responses

**Fig. 9:**
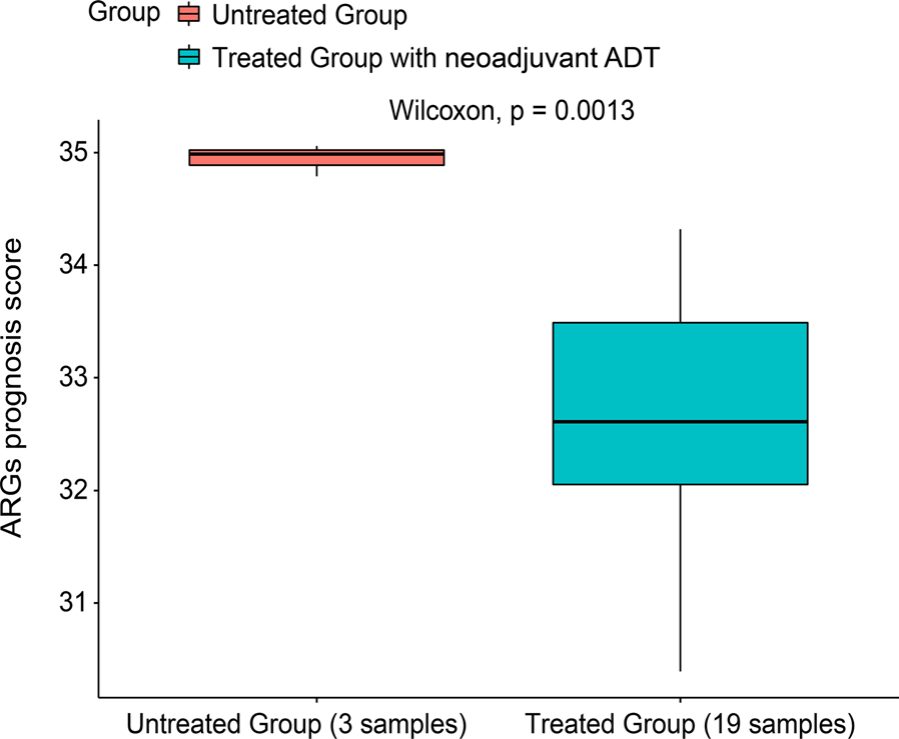
The evaluation of a neoadjuvant androgen deprivation therapy (ADT) response

The endocrine therapy has been one of the standard treatment strategies to improve the clinical outcomes of PCa patients. Remarkably, it was found that the ARGs signature score of the primary prostate cancer patients from the GSE102124 dataset was significantly downregulated (p = 0.0013, Fig. 9) after receiving the conjunction treatment of leuprolide, abiraterone acetate, and prednisone, which was a neoadjuvant ADT. However, it was observed that LHRH agonist and orchiectomy treatments had no influence on the regulation of the ARGs signature score of CRPC patients from the GSE74685 dataset (p > 0.05, Additional file 4: Fig. S4).

**Fig. 10:**
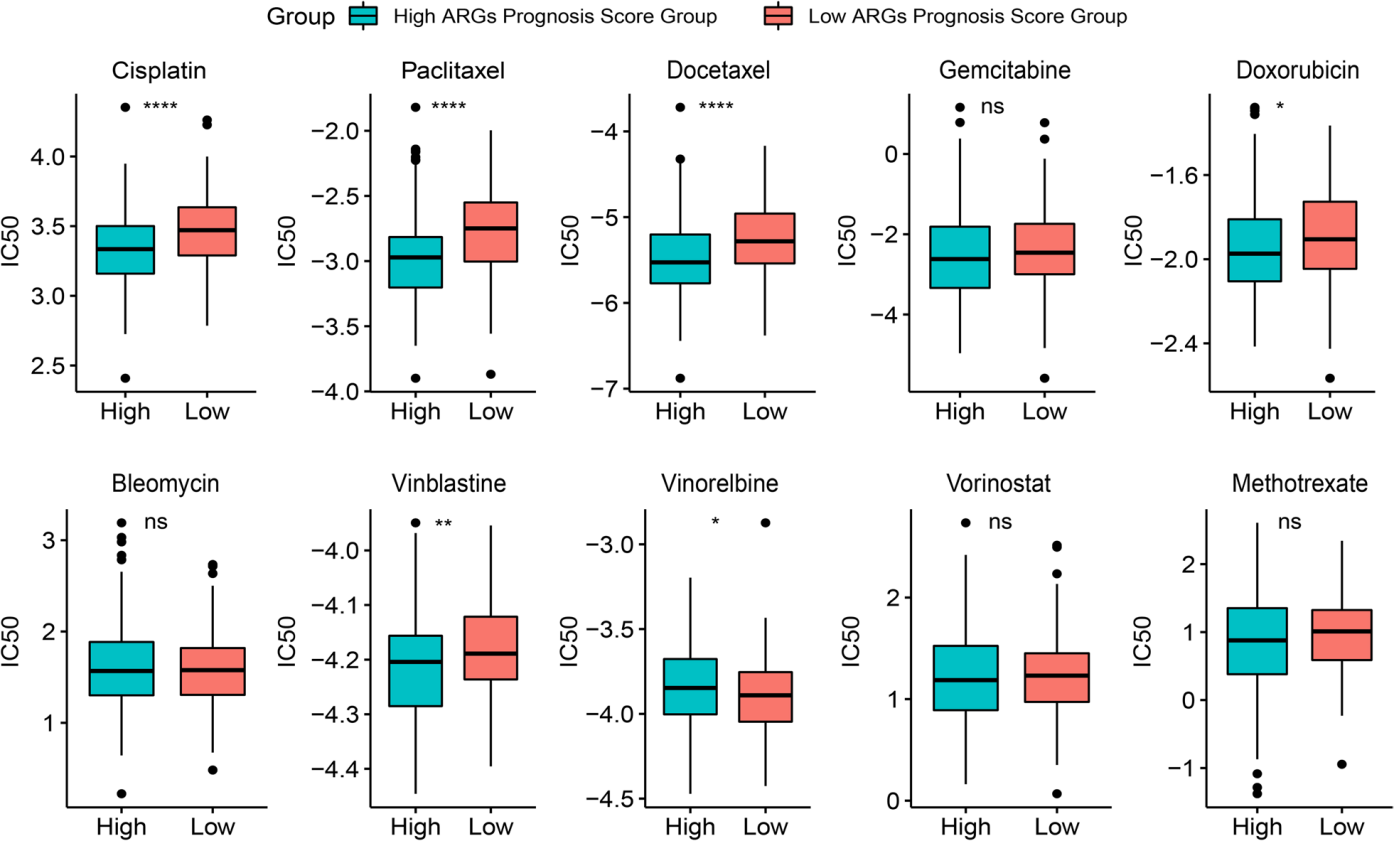
The evaluation of the selected chemotherapy drugs between high- and low-ARGs signature score groups. (*p < 0.05, **p < 0.01, ****p < 0.0001)

The data from the GDSC database were utilized to predict the possible responses to some traditional chemotherapy drugs in the cancer. It could be observed that there was a statistically significant difference of IC50 among six chemotherapy drugs between the high- and low-ARGs signature score groups. Moreover, PRAD patients with the high ARGs signature score seemed to be more sensitive to five identified chemotherapy drugs (cisplatin, paclitaxel, docetaxel, vinblastine and doxorubicin, p < 0.05, Fig. 10). Nevertheless, PRAD patients with low ARGs signature score seemed to be more sensitive to vinorelbine (Fig. 10). Of note, it was further found in PC3 PCa cell line from the GSE140440 dataset that high ARGs signature score group was significantly correlated with the sensitivity of docetaxel (p < 0.0001, Additional file 5: Fig. S5). In addition, correlation analysis revealed that in the high ARGs signature score group not all signature-related ARGs were associated with sensitivity to the five identified chemotherapy drugs, while only *LRPAP1* was significantly correlated with sensitivity to all these five chemotherapy drugs and positively correlated with cisplatin and doxorubicin but negatively correlated with paclitaxel, docetaxel, and vinblastine (p < 0.05, Additional file 6: Fig. S6). Whereas, it could be obviously observed that most ARGs in signature were significantly correlated with sensitivity to the six identified chemotherapy drugs (p < 0.05, Additional file 6: Fig. S6).

### IHC analysis of ARGs protein expression in human PRAD samples

**Fig. 11:**
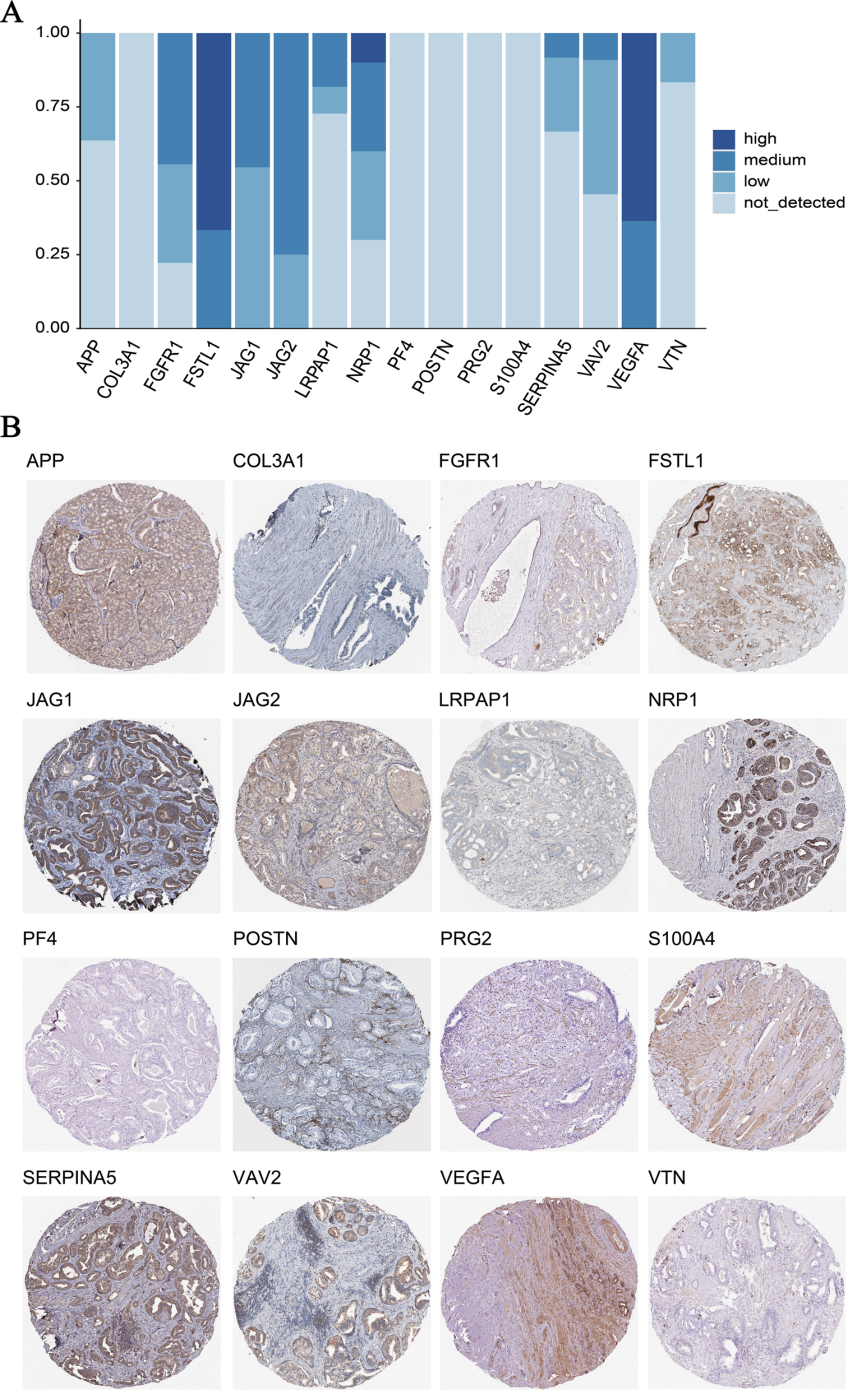
The immunohistochemistry (IHC) analysis. **A** The collection of IHC staining information of ARGs involved in the signature. **B** The representative IHC staining of signature-related ARGs

Additionally, IHC data were retrieved from the HPA database to investigate the signature-related ARGs at protein level. It was found that 4/11, 7/9, 12/12, 11/11, 12/12, 3/11, 7/10, 4/12, 6/11, 11/11, and 2/12 PRAD samples expressed APP, FGFR1, FSTL1, JAG1, JAG2, LRPAP1, NRP1, SERPINA5, VAV2, VEGFA, and VTN, respectively (Fig. 11A). Of note, it was observed that PRAD samples had the protein expression of these 11 ARGs all located in the cytoplasm and/or membrane by the IHC analysis (Fig. 11B). Whereas, COL3A1, PF4, POSTN, PRG2, and S100A4 were not detected in any PRAD samples (Fig. 11A&B). Due to the lack of IHC staining of CXCL6, OLR1, and LPL in PRAD samples, of which the protein expression was not estimated.

## Discussions

Although emerging strategy of anti-angiogenesis has been validated as an effective treatment for multiple solid tumors including renal carcinoma, lung cancer and stomach cancer (Schmidt and Carmeliet 2011; Sarkar et al. 2015), unfortunately, it did not exhibit promising clinical efficacy as expected in the PCa. On the other side, the integrated role of ARGs in predicting the prognosis of PCa has been neglected and scarcely studied. The present study offered a deep insight of the integrated function of angiogenesis in the prognosis of PCa.

The angiogenesis-related gene set, namely the Hallmark_Angiogenesis gene set from Molecular Signatures Database, was selected to establish the signature. The hallmark gene set has reduced noise and redundancy, as well as controlled false discoveries by identifying gene set overlaps and analyzing coordinate expression (Liberzon et al. 2015). We investigated the specific association between these ARGs and DFS of PRAD and found that patients with altered expression of ARG(s) were significantly correlated with poorer DFS, compared to those without any alterations. However, no significant difference was observed in the DFS of groups with or without ARG genetic mutations. Then, we identified a total of 23 ARGs that significantly correlated with PCa patients’ survival. In concordance with previous studies which investigate the ARG solely in prostate cancer, genes including *NRP1* (Tse et al. 2017), *JAG2* (Kwon et al. 2016), *COL5A2* (Ren et al. 2021), *POSTN* (Cattrini et al. 2018), *FSTL1* (Zhao et al. 2019), *PF4* (Baselga et al. 2008), *JAG1* (Terada et al. 2014), *COL3A1* (Angel et al. 2020), *VCAN* (Asano et al. 2017), *VEGFA* (Zhan et al. 2013), *SPP1* (Pang et al. 2019), *VTN* (Niu et al. 2016), and *S100A4* (Ganaie et al. 2020) were identified as unfavorable prognosis-related biomarkers in prostate cancer. Meanwhile, the prognosis roles of *APP* (Takayama et al. 2009) and *FGFR1* (Yang et al. 2013b) were controversial with previous studies, which needed further validation and study. Notably, genes including *PRG2*, *VAV2*, *LPL*, *OLR1*, *SERPINA5*, *CXCL6*, *LRPA1*, and *KCNJ8* have not been identified to be correlated with the prognosis of PCa before, meanwhile, it was also found that *CXCL6*, *KCNJ8*, *LPL*, and *SERPINA5* were upregulated in the tumor samples by comparison with normal samples. Thus, it is worthy to investigate the specific biologial function of these genes in the carcinogenesis and development of PCa in the further study.

Based on the expression profiling of the ARGs gene set, the signature (a 19-ARG signature) was developed, which had the ability to predict the prognosis of PRAD patients. Meanwhile, the result of clinical association analysis demonstrated that the higher ARGs signature score indicated the advanced clinical status as well as the higher expression of clinically used tumor angiogenesis markers (*CD34* & *CD105*), which could further help clinicians promote the PRAD management. In addition, a prognostic nomogram based on the combination of ARGs signature score and two clinical features (Gleason score & T stage) was established with the high AUC values of 0.82, 0.83, and 0.83 for the DFS at 1, 3, and 5 years, respectively, demonstrating more reliable and stable capacity of nomogram to predict prognosis of PRAD patients and which was also verified in other two independent PRAD patient cohorts, by comparison with the performance of the ARGs signature solely in prognosis prediction. Thus, the ARGs signature-based prognostic nomogram was highly recommended for future clinical applications. Previous studies had demonstrated the influence of angiogenesis on tumor growth and progression (Katayama et al. 2019; Carmeliet and Jain 2000; Ramjiawan et al. 2017; Pavlakovic et al. 2001; Schmidt and Carmeliet 2011; Baselga et al. 2008; Goos et al. 2016; Sbiera et al. 2017; Wei et al. 2020; Dunne et al. 2006; Townsend et al. 2019; Javaherian and Lee 2011; Lin et al. 2003; Maurer et al. 2006; Yang et al. 2020). Moreover, inhibiting angiogenesis has emerged as an effective therapeutic strategy for most solid tumors. But past researches of ARGs or angiogenesis-related pathways in PCa are limited, most studies are intrigued by the VEGF-related pathways and develop new medicines for the VEGF-related targets in PCa (Eisermann and Fraizer 2017; Sarkar et al. 2020). However, very few novel therapies showed significant improvement on the survival of PCa patients (McKay et al. 2016; Michaelson et al. 2014; Horti et al. 2009; Tannock et al. 2013). Endocrine therapy is still the common treatment for advanced PCa patients by far, including ADT and novel hormone treatments, etc. (Zhu and Ye 2020; Tietz and Dehm 2020; Mollica et al. 2021). Nevertheless, endocrine therapies give rise to CRPC (Eisermann and Fraizer 2017). Whereas, the integrated angiogenesis activity has been found to be correlated with PRAD progression, thus the identified 19 ARGs instead of one single ARG could become the molecularly therapeutic targets for PRAD.

Hallmark gene set analysis and KEGG pathway enrichment analysis showed significant differences in the biological processes of patients between the high and low ARGs signature score group. Interestingly, the E2F targets, G2M checkpoint pathways, and cell cycle pathways positively correlated with patients in high ARGs signature score group, and these signaling pathway were all vital to the tumor progression and metastasis (Löbrich and Jeggo 2007). For the targeted therapy, cell cycle pathway inhibitors could be used to improve the prognosis of PRAD. Besides, the functional analyses further showed the significant differences of cell metabolism, including fatty acid metabolism, bile acid metabolism and peroxide metabolism/peroxisome, between the high and low ARGs signature score group. As was reported previously, the aberrant regulation of fatty acid metabolism, bile acid metabolism and peroxide metabolism would cause the accumulation of reactive oxygen species (ROS) and/or peroxide, leading to the oxidative stress (Sies and Jones 2020). Generally, oxidative eustress promoted the activities of angiogenesis and cell proliferation, differentiation, and migration, but oxidative distress contributed the inflammation, fibrogenesis, and tumor metastasis (Sies and Jones 2020; Hayes et al. 2020). Therefore, specific targeted drugs in the regulation of redox signaling pathways could be suggested for the treatment of PRAD, while which needs to be further explored. We also investigated the infiltration of immune cell, and found that patients in the high ARGs signature score group had significantly higher ESTIMATE score correlated with the lower tumor purity (Yoshihara et al. 2013b), but who had significantly higher immune score as well as higher expression of immune checkpoint genes, suggesting that the immune therapies could be more effective for the high-risk PRAD patients, however, the higher presence of Tregs in patients with high ARGs signature may hinder them from benefits from immune checkpoint inhibitors.

As demonstrated, neoadjuvant ADT combining leuprolide, abiraterone acetate, and prednisone had the capacity of diminishing the ARGs signature score, which predicted the relationship between ARGs and PCa, suggesting this signature had the potential to serve as a response-related biomarker (Sowalsky et al. 2018). However, it was also found that LHRH agonists and orchiectomy exerted no significant difference on the ARGs signature score in CRPC patients. These differences may attribute from the involved patients were encountered with different status of disease and different treatments. On the other hand, the ARGs signature score could also predict the efficacy of treatments, which would be of great merit to the management of PCa. In addition, the chemotherapy was often used in treating advanced PCa as well. Findings in the evaluation of chemotherapy responses demonstrated that relatively advanced patients who had the high ARGs signature score were more suitable to take the treatment with cisplatin, paclitaxel, docetaxel, vinblastine and doxorubicin, all of which had the ability to inhibit angiogenesis in prostate tumors (Shankar et al. 2005; Quesada et al. 2005; Karashima et al. 2002; Kumar et al. 2005; Reiner 2006). Among them, docetaxel is the standard care for treating the mCRPC patients, and as suggested in previous study, it was also recommended to be early used for metastatic hormone-sensitive PCa (mHSPC) (Markowski and Carducci 2017). However, there were few experimental studies demonstrating whether these 19 ARGs were collectively associated with sensitivity to the five identified chemo drugs except studies that docetaxel could markedly suppress the expression of *VEGFA* (Zhu et al. 2014), and the *VEGFA-VEGFR2* signaling pathway could be regulated when receiving the chemo drugs of cisplatin or paclitaxel in prostate cancer (Yun et al. 2021). Therefore, in the future work it is necessary to figure out if these 19 ARGs were collectively associated with the sensitivity to the identified chemotherapy drugs in clinical trials. The established ARGs signature may have the potential to distinct the mHSPC patients who may have more clinical benefit to ADT with docetaxel than ADT alone or combination with novel hormone treatments, which merits further validation. Overall, the high ARGs signature score can indicate the relatively advanced PRAD, which is of great value for clinicians to make better decisions for the management of PRAD.

Though we have established a robust and meaningful tool in PCa based on the ARGs, there were some limitations. Firstly, our study was based on the public database, thus it would be necessary to further verify these findings in prospective cohort from our hospital. In addition, the roles of angiogenesis involved in the progression of PCa are also needed to be explored in the mouse model and/or the cell lines.

## Conclusions

This is the first reported angiogenesis-related multigene signature in PCa. The present study determined a 19-ARG signature scoring model and a prognostic nomogram, which were very robust, stable and reliable for predicting the clinic outcomes of PRAD patients. In addition, this defined 19 ARGs expression could also be promoted as a decision-making tool for selecting treatment strategy to improve the outcomes of PRAD patients.

## Supplementary Information

**Additional file 1. Fig. S1.** The association between ARGs signature score and the expression of common tumor angiogenesis markers of CD34 & CD105.

**Additional file 2. Fig. S2.** The comparison between ARGs signature and each ARG in prognosis prediction via the receiver operating characteristic curve (ROC) analysis.

**Additional file 3. Fig. S3.** The evaluation of nomogram in disease-free survival (DFS) prediction the receiver operating characteristic curve (ROC) analysis in GSE40272 and PRAD_MSKCC.

**Additional file 4. Fig. S4.** The comparison of ARGs signature score between castration-resistant prostate cancer (CRPC) patients receiving luteinizing hormone-releasing hormone (LHRH) agonist or orchiectomy treatments, and some not receiving any therapies.

**Additional file 5.** The validation of ARGs signature correlated with the sensitivity to docetaxel in PC3 PCa cell line in GSE140440.

**Additional file 6. Fig. S6.** The correlation analysis between each ARG expression and sensitivity to chemotherapy drugs, including cisplatin, paclitaxel, docetaxel, gemcitabine, doxorubicin, bleomycin, vinblastine, vinorelbine, vorinostat, methotrexate.

**Additional file 7: Table S1.** The differentiation analysis in clinical characteristics of PRAD samples between the TCGA-PRAD cohort and GSE40272 dataset.

**Additional file 8: Table S2.** The differentiation analysis in clinical characteristics of PRAD samples between the TCGA-PRAD cohort and PRAD_MSKCC cohort.

**Additional file 9. Table S3.** The differentially expressed ARGs between tumor and normal tissue samples.

**Additional file 10. Table S4.** The comparison of altered genes between high- and low- ARGs signature score groups.

**Additional file 11. Table S5.** The comparsion of prevalently altered genes between high- and low- ARGs signaturescore groups.

**Additional file 12. Table S6.** The Hallmark gene set enrichment analysis underlying the expression of each ARGs involved in the signature.

**Additional file 13. Table S7.** The KEGG pathway enrichment analysis underlying the expression of each ARGs involved in the signature.

**Additional file 14. Table S8.** The Hallmark gene set enrichment analysis underlying the ARGs signature.

**Additional file 15. Table S9.** The KEGG pathway enrichment analysis underlying the ARGs signature.

## Data Availability

The datasets analyzed in the present study was from the public database. The data can be downloaded from: https://www.cbioportal.org/study/summary?id=prad_tcga, https://gdc-hub.s3.us-east-1.amazonaws.com/download/TCGA-PRAD.htseq_counts.tsv.gz, https://www.ncbi.nlm.nih.gov/geo/, http://www.gsea-msigdb.org/gsea/msigdb/, https://www.cancerrxgene.org/.

## Abbreviations

ARGs: Angiogenesis-related genes
PCa: Prostate cancer
PRAD: Prostate adenocarcinoma
TCGA: The Cancer Genome Atlas
DFS: Disease-free survival
LASSO: Least absolute shrinkage and selection operator
ROC: Receiver operating characteristic curve
AUC: Area under receiver operating characteristic curve
ADT: Androgen deprivation therapy
NGS: Next generation sequencing
TME: Tumor microenvironment
GEO: Gene expression omnibus
MSigDB: Molecular signatures database
KEGG: Kyoto Encyclopedia of Genes and Genomes
CIBERSORT: Cell type Identification By Estimating Relative Subsets Of known RNA Transcripts
ESTIMATE: Estimation of STromal and Immune cells in MAlignant Tumours using Expression data
CRPC: Castration-resistant prostate cancer
LHRH: Luteinizing hormone-releasing hormone
GDSC: Genomics of drug sensitivity in cancer
IC50: Half-maximal inhibitory concentration
PSA: Prostate specific antigen
C-index: Concordance index
DDR: DNA damage response
mHSPC: Metastatic hormone-sensitive prostate cancer
IHC: Immunohistochemistry

## References

[CR1] Angel P, Spruill L, Jefferson M, Bethard J, Ball L, Hughes-Halbert C, Drake R (2020). Zonal regulation of collagen-type proteins and posttranslational modifications in prostatic benign and cancer tissues by imaging mass spectrometry. Prostate.

[CR2] Armenia J, Wankowicz SAM, Liu D, Gao J, Kundra R, Reznik E, Chatila WK, Chakravarty D, Han GC, Coleman I (2018). The long tail of oncogenic drivers in prostate cancer. Nat Genet.

[CR3] Asano K, Nelson C, Nandadasa S, Aramaki-Hattori N, Lindner D, Alban T, Inagaki J, Ohtsuki T, Oohashi T, Apte S (2017). Stromal versican regulates tumor growth by promoting angiogenesis. Sci Rep.

[CR4] Baselga J, Rothenberg M, Tabernero J, Seoane J, Daly T, Cleverly A, Berry B, Rhoades S, Ray C, Fill J (2008). TGF-beta signalling-related markers in cancer patients with bone metastasis. Biomarkers Biochem Indic Expo Response Suscept Chem.

[CR5] Bertoli G, Cava C, Castiglioni I (2016). MicroRNAs as biomarkers for diagnosis, prognosis and theranostics in prostate cancer. Int J Mol Sci.

[CR6] Carmeliet P, Jain R (2000). Angiogenesis in cancer and other diseases. Nature.

[CR7] Cattrini C, Rubagotti A, Nuzzo P, Zinoli L, Salvi S, Boccardo S, Perachino M, Cerbone L, Vallome G, Latocca M (2018). Overexpression of periostin in tumor biopsy samples is associated with prostate cancer phenotype and clinical outcome. Clin Genitourin Cancer.

[CR8] Chen S, Zhu G, Yang Y, Wang F, Xiao Y, Zhang N, Bian X, Zhu Y, Yu Y, Liu F (2021). Single-cell analysis reveals transcriptomic remodellings in distinct cell types that contribute to human prostate cancer progression. Nat Cell Biol.

[CR9] Chun F, Karakiewicz P, Briganti A, Gallina A, Kattan M, Montorsi F, Huland H, Graefen M (2006). Prostate cancer nomograms: an update. Eur Urol.

[CR10] Cucchiara V, Cooperberg M, Dall'Era M, Lin D, Montorsi F, Schalken J, Evans C (2018). Genomic markers in prostate cancer decision making. Eur Urol.

[CR11] Dell'Oglio P, Suardi N, Boorjian S, Fossati N, Gandaglia G, Tian Z, Moschini M, Capitanio U, Karakiewicz P, Montorsi F (2016). Predicting survival of men with recurrent prostate cancer after radical prostatectomy. Eur J Cancer (oxford, England: 1990).

[CR12] Dunne J, Cullmann C, Ritter M, Soria N, Drescher B, Debernardi S, Skoulakis S, Hartmann O, Krause M, Krauter J (2006). siRNA-mediated AML1/MTG8 depletion affects differentiation and proliferation-associated gene expression in t(8;21)-positive cell lines and primary AML blasts. Oncogene.

[CR13] Eisermann K, Fraizer G (2017). The Androgen Receptor and VEGF: mechanisms of androgen-regulated angiogenesis in prostate cancer. Cancers.

[CR14] Eure G, Germany R, Given R, Lu R, Shindel A, Rothney M, Glowacki R, Henderson J, Richardson T, Goldfischer E (2017). Use of a 17-gene prognostic assay in contemporary urologic practice: results of an interim analysis in an observational cohort. Urology.

[CR15] Faisal F, Lotan T (2020). The genomic and molecular pathology of prostate cancer: clinical implications for diagnosis, prognosis, and therapy. Adv Anat Pathol.

[CR16] Feng R, Zong Y, Cao S, Xu R (2019). Current cancer situation in China: good or bad news from the 2018 Global Cancer Statistics?. Cancer Commun (London, England).

[CR17] Ganaie A, Mansini A, Hussain T, Rao A, Siddique H, Shabaneh A, Ferrari M, Murugan P, Klingelhöfer J, Wang J (2020). S100A4Anti-S100A4 antibody therapy is efficient in treating aggressive prostate cancer and reversing immunosuppression: serum and biopsy as a clinical predictor. Mol Cancer Ther.

[CR18] Garnett M, Edelman E, Heidorn S, Greenman C, Dastur A, Lau K, Greninger P, Thompson I, Luo X, Soares J (2012). Systematic identification of genomic markers of drug sensitivity in cancer cells. Nature.

[CR19] Goos J, de Cuba E, Coupé V, Diosdado B, Delis-Van Diemen P, Karga C, Beliën J, Menke-Van der Houven van Oordt C, Geldof A, Meijer G (2016). Glucose Transporter 1 (SLC2A1) and vascular endothelial growth factor A (VEGFA) predict survival after resection of colorectal cancer liver metastasis. Ann Surg.

[CR20] Hayes J, Dinkova-Kostova A, Tew K (2020). Oxidative stress in cancer. Cancer Cell.

[CR21] Horti J, Widmark A, Stenzl A, Federico M, Abratt R, Sanders N, Pover G, Bodrogi I (2009). A randomized, double-blind, placebo-controlled phase II study of vandetanib plus docetaxel/prednisolone in patients with hormone-refractory prostate cancer. Cancer Biother Radiopharm.

[CR22] Iorio F, Knijnenburg T, Vis D, Bignell G, Menden M, Schubert M, Aben N, Gonçalves E, Barthorpe S, Lightfoot H (2016). A landscape of pharmacogenomic interactions in cancer. Cell.

[CR23] Javaherian K, Lee T, TjinThamSjin R, Parris G, Hlatky L (2011). Two endogenous antiangiogenic inhibitors, endostatin and angiostatin, demonstrate biphasic curves in their antitumor profiles. Dose-Response.

[CR24] Karashima T, Sweeney P, Slaton J, Kim S, Kedar D, Izawa J, Fan Z, Pettaway C, Hicklin D, Shuin T (2002). Inhibition of angiogenesis by the antiepidermal growth factor receptor antibody ImClone C225 in androgen-independent prostate cancer growing orthotopically in nude mice. Clin Cancer Res.

[CR25] Katayama Y, Uchino J, Chihara Y, Tamiya N, Kaneko Y, Yamada T, Takayama K (2019). Tumor neovascularization and developments in therapeutics. Cancers.

[CR26] Kumar P, Benedict R, Urzua F, Fischbach C, Mooney D, Polverini P (2005). Combination treatment significantly enhances the efficacy of antitumor therapy by preferentially targeting angiogenesis. Lab Investig J Tech Methods Pathol.

[CR27] Kumar A, Coleman I, Morrissey C, Zhang X, True L, Gulati R, Etzioni R, Bolouri H, Montgomery B, White T (2016). Substantial interindividual and limited intraindividual genomic diversity among tumors from men with metastatic prostate cancer. Nat Med.

[CR28] Kwon O, Zhang L, Wang J, Su Q, Feng Q, Zhang X, Mani S, Paulter R, Creighton C, Ittmann M (2016). Notch promotes tumor metastasis in a prostate-specific Pten-null mouse model. J Clin Investig.

[CR29] Lam D, Clark S, Stirzaker C, Pidsley R (2020). Advances in Prognostic Methylation Biomarkers for Prostate Cancer. Cancers.

[CR30] Liberzon A, Birger C, Thorvaldsdóttir H, Ghandi M, Mesirov J, Tamayo P (2015). the molecular signatures database (MSigDB) hallmark gene set collection. Cell Syst.

[CR31] Lin C, Leu S, Chen N, Tebeau C, Lin S, Yeung C, Lau L (2003). CCN3 (NOV) is a novel angiogenic regulator of the CCN protein family. J Biol Chem.

[CR32] Löbrich M, Jeggo P (2007). The impact of a negligent G2/M checkpoint on genomic instability and cancer induction. Nat Rev Cancer.

[CR33] Markowski M, Carducci M (2017). Early use of chemotherapy in metastatic prostate cancer. Cancer Treat Rev.

[CR34] Maurer A, Zhou B, Han Z (2006). Roles of platelet factor 4 in hematopoiesis and angiogenesis. Growth Factors (chur, Switzerland).

[CR35] McKay R, Zurita A, Werner L, Bruce J, Carducci M, Stein M, Heath E, Hussain A, Tran H, Sweeney C (2016). A randomized phase II trial of short-course androgen deprivation therapy with or without bevacizumab for patients with recurrent prostate cancer after definitive local therapy. J Clin Oncol.

[CR36] Melegh Z, Oltean S (2019). Targeting Angiogenesis in Prostate Cancer. Int J Molec Sci.

[CR37] Michaelson M, Oudard S, Ou Y, Sengeløv L, Saad F, Houede N, Ostler P, Stenzl A, Daugaard G, Jones R (2014). Randomized, placebo-controlled, phase III trial of sunitinib plus prednisone versus prednisone alone in progressive, metastatic, castration-resistant prostate cancer. J Clin Oncol.

[CR38] Mollica V, Rizzo A, Rosellini M, Marchetti A, Ricci A, Cimadamore A, Scarpelli M, Bonucci C, Andrini E, Errani C, Santoni M, Montironi R, Massari F (2021). Bone targeting agents in patients with metastatic prostate cancer: state of the art. Cancers.

[CR39] Niu Y, Zhang L, Bi X, Yuan S, Chen P (2016). Evaluation of vitronectin expression in prostate cancer and the clinical significance of the association of vitronectin expression with prostate specific antigen in detecting prostate cancer. Urol J.

[CR40] Pang X, Xie R, Zhang Z, Liu Q, Wu S, Cui Y (2019). Identification of SPP1 as an extracellular matrix signature for metastatic castration-resistant prostate cancer. Front Oncol.

[CR41] Pavlakovic H, Havers W, Schweigerer L (2001). Multiple angiogenesis stimulators in a single malignancy: implications for anti-angiogenic tumour therapy. Angiogenesis.

[CR42] Quesada A, Nelius T, Yap R, Zaichuk T, Alfranca A, Filleur S, Volpert O, Redondo J (2005). In vivo upregulation of CD95 and CD95L causes synergistic inhibition of angiogenesis by TSP1 peptide and metronomic doxorubicin treatment. Cell Death Differ.

[CR43] Rajabi M, Mousa S (2017). The role of angiogenesis in cancer treatment. Biomedicines.

[CR44] Ramjiawan R, Griffioen A, Duda D (2017). Anti-angiogenesis for cancer revisited: is there a role for combinations with immunotherapy?. Angiogenesis.

[CR45] Rebello R, Oing C, Knudsen K, Loeb S, Johnson D, Reiter R, Gillessen S, Van der Kwast T, Bristow R (2021). Prostate cancer. Nat Rev Dis Prim.

[CR46] Reiner T, de las Pozas A, Perez-Stable C (2006). Sequential combinations of flavopiridol and docetaxel inhibit prostate tumors, induce apoptosis, and decrease angiogenesis in the Ggamma/T-15 transgenic mouse model of prostate cancer. Prostate.

[CR47] Ren X, Chen X, Fang K, Zhang X, Wei X, Zhang T, Li G, Lu Z, Song N, Wang S (2021). COL5A2 promotes proliferation and invasion in prostate cancer and is one of seven Gleason-related genes that predict recurrence-free survival. Front Oncol.

[CR48] Sarkar C, Chakroborty D, Dasgupta P, Basu S (2015). Dopamine is a safe antiangiogenic drug which can also prevent 5-fluorouracil induced neutropenia. Int J Cancer.

[CR49] Sarkar C, Goswami S, Basu S, Chakroborty D (2020). Angiogenesis inhibition in prostate cancer: an update. Cancers.

[CR50] Sauerbrei W, Royston P, Binder H (2007). Selection of important variables and determination of functional form for continuous predictors in multivariable model building. Stat Med.

[CR51] Sbiera S, Sbiera I, Ruggiero C, Doghman-Bouguerra M, Korpershoek E, de Krijger R, Ettaieb H, Haak H, Volante M, Papotti M (2017). Assessment of VAV2 expression refines prognostic prediction in adrenocortical carcinoma. J Clin Endocrinol Metab.

[CR52] Schmidt T, Carmeliet P (2011). Angiogenesis: a target in solid tumors, also in leukemia?. Hematol Am Soc Hematol Educ Program.

[CR53] Schnepp PM, Shelley G, Dai J, Wakim N, Jiang H, Mizokami A, Keller ET (2020). Single-cell transcriptomics analysis identifies nuclear protein 1 as a regulator of docetaxel resistance in prostate cancer cells. Mol Cancer Res.

[CR54] Shankar S, Chen X, Srivastava R (2005). Effects of sequential treatments with chemotherapeutic drugs followed by TRAIL on prostate cancer in vitro and in vivo. Prostate.

[CR55] Shore N, Kella N, Moran B, Boczko J, Bianco F, Crawford E, Davis T, Roundy K, Rushton K, Grier C (2016). Impact of the cell cycle progression test on physician and patient treatment selection for localized prostate cancer. J Urol.

[CR56] Sies H, Jones D (2020). Reactive oxygen species (ROS) as pleiotropic physiological signalling agents. Nat Rev Mol Cell Biol.

[CR57] Sowalsky A, Ye H, Bhasin M, Van Allen E, Loda M, Lis R, Montaser-Kouhsari L, Calagua C, Ma F, Russo J (2018). Neoadjuvant-intensive androgen deprivation therapy selects for prostate tumor foci with diverse subclonal oncogenic alterations. Cancer Res.

[CR58] Spratt D, Yousefi K, Deheshi S, Ross A, Den R, Schaeffer E, Trock B, Zhang J, Glass A, Dicker A (2017). Individual patient-level meta-analysis of the performance of the decipher genomic classifier in high-risk men after prostatectomy to predict development of metastatic disease. J Clin Oncol.

[CR59] Strohmeyer D, Rössing C, Strauss F, Bauerfeind A, Kaufmann O, Loening S (2000). Tumor angiogenesis is associated with progression after radical prostatectomy in pT2/pT3 prostate cancer. Prostate.

[CR60] Takayama K, Tsutsumi S, Suzuki T, Horie-Inoue K, Ikeda K, Kaneshiro K, Fujimura T, Kumagai J, Urano T, Sakaki Y (2009). Amyloid precursor protein is a primary androgen target gene that promotes prostate cancer growth. Cancer Res.

[CR61] Tannock I, Fizazi K, Ivanov S, Karlsson C, Fléchon A, Skoneczna I, Orlandi F, Gravis G, Matveev V, Bavbek S (2013). Aflibercept versus placebo in combination with docetaxel and prednisone for treatment of men with metastatic castration-resistant prostate cancer (VENICE): a phase 3, double-blind randomised trial. Lancet Oncol.

[CR62] Terada N, Shiraishi T, Zeng Y, Aw-Yong K, Mooney S, Liu Z, Takahashi S, Luo J, Lupold S, Kulkarni P (2014). Correlation of Sprouty1 and Jagged1 with aggressive prostate cancer cells with different sensitivities to androgen deprivation. J Cell Biochem.

[CR63] Tietz K, Dehm S (2020). Androgen receptor variants: RNA-based mechanisms and therapeutic targets. Hum Mol Genet.

[CR64] Townsend M, Ence Z, Felsted A, Parker A, Piccolo S, Robison R, O'Neill K (2019). Potential new biomarkers for endometrial cancer. Cancer Cell Int.

[CR65] Tse B, Volpert M, Ratther E, Stylianou N, Nouri M, McGowan K, Lehman M, McPherson S, Roshan-Moniri M, Butler M (2017). Neuropilin-1 is upregulated in the adaptive response of prostate tumors to androgen-targeted therapies and is prognostic of metastatic progression and patient mortality. Oncogene.

[CR66] Wang H, Lengerich B, Aragam B, Xing E (2019). Precision Lasso: accounting for correlations and linear dependencies in high-dimensional genomic data. Bioinformatics (oxford, England).

[CR67] Wei Y, Guo S, Tang J, Wen J, Wang H, Hu X, Gu Q (2020). MicroRNA-19b-3p suppresses gastric cancer development by negatively regulating neuropilin-1. Cancer Cell Int.

[CR68] Yang W, Soares J, Greninger P, Edelman E, Lightfoot H, Forbes S, Bindal N, Beare D, Smith J, Thompson I (2013). Genomics of drug sensitivity in cancer (GDSC): a resource for therapeutic biomarker discovery in cancer cells. Nucleic Acids Res.

[CR69] Yang F, Zhang Y, Ressler S, Ittmann M, Ayala G, Dang T, Wang F, Rowley D (2013). FGFR1 is essential for prostate cancer progression and metastasis. Cancer Res.

[CR70] Yang Y, Lu T, Li Z, Lu S (2020). FGFR1 regulates proliferation and metastasis by targeting CCND1 in FGFR1 amplified lung cancer. Cell Adhes Migr.

[CR71] Yin L, Li J, Wang J, Pu T, Wei J, Li Q, Wu B (2021). MAOA promotes prostate cancer cell perineural invasion through SEMA3C/PlexinA2/NRP1-cMET signaling. Oncogene.

[CR72] Yoshihara K, Shahmoradgoli M, Martínez E, Vegesna R, Kim H, Torres-Garcia W, Treviño V, Shen H, Laird P, Levine D (2013). Inferring tumour purity and stromal and immune cell admixture from expression data. Nat Commun.

[CR73] Yoshihara K, Shahmoradgoli M, Martínez E, Vegesna R, Kim H, Torres-Garcia W, Treviño V, Shen H, Laird PW, Levine DA (2013). Inferring tumour purity and stromal and immune cell admixture from expression data. Nat Commun.

[CR74] Yu G, Wang L, Han Y, He Q (2012). clusterProfiler: an R package for comparing biological themes among gene clusters. OMICS.

[CR75] Yun J, Lee S, Chun S, Lee K, Kim J, Kim H (2021). Comprehensive analysis of oncogenic signatures and consequent repurposed drugs in TMPRSS2:ERG fusion-positive prostate cancer. Clin Transl Med.

[CR76] Zhan P, Ji Y, Yu L (2013). VEGF is associated with the poor survival of patients with prostate cancer: a meta-analysis. Transl Androl Urol.

[CR77] Zhao Y, Ou Q, Deng Y, Peng J, Li C, Li J, Zhao Q, Qiu M, Wan D, Fang Y (2019). Determination of follistatin-like protein 1 expression in colorectal cancer and its association with clinical outcomes. Ann Transl Med.

[CR78] Zhao Y, Cai C, Zhang M, Shi L, Wang J, Zhang H, Ma P, Li S (2021). Ephrin-A2 promotes prostate cancer metastasis by enhancing angiogenesis and promoting EMT. J Cancer Res Clin Oncol.

[CR79] Zhu Y, Ye D (2020). Chinese expert consensus on the diagnosis and treatment of castration-resistant prostate cancer (2019 update). Cancer Manag Res.

[CR80] Zhu S, Oremo J, Li S, Zhen M, Tang Y, Du Y (2014). Synergistic antitumor activities of docetaxel and octreotide associated with apoptotic-upregulation in castration-resistant prostate cancer. PLoS ONE.

[CR81] Zhuo Y, Liu Z, Wan S, Cai Z, Xie J, Cai Z, Song S, Wan Y, Hua W, Zhong W (2018). Enhanced expression of SRPK2 contributes to aggressive progression and metastasis in prostate cancer. Biomed Pharmacother.

