# Supplementary figures and images for: The novel transcriptomic signature of angiogenesis predicts clinical outcome, tumor microenvironment and treatment response for prostate adenocarcinoma
Source: Mol Med. 2022 Jul 14;28:78. doi: 10.1186/s10020-022-00504-6 (PMC9284787; doi:10.1186/s10020-022-00504-6)

A

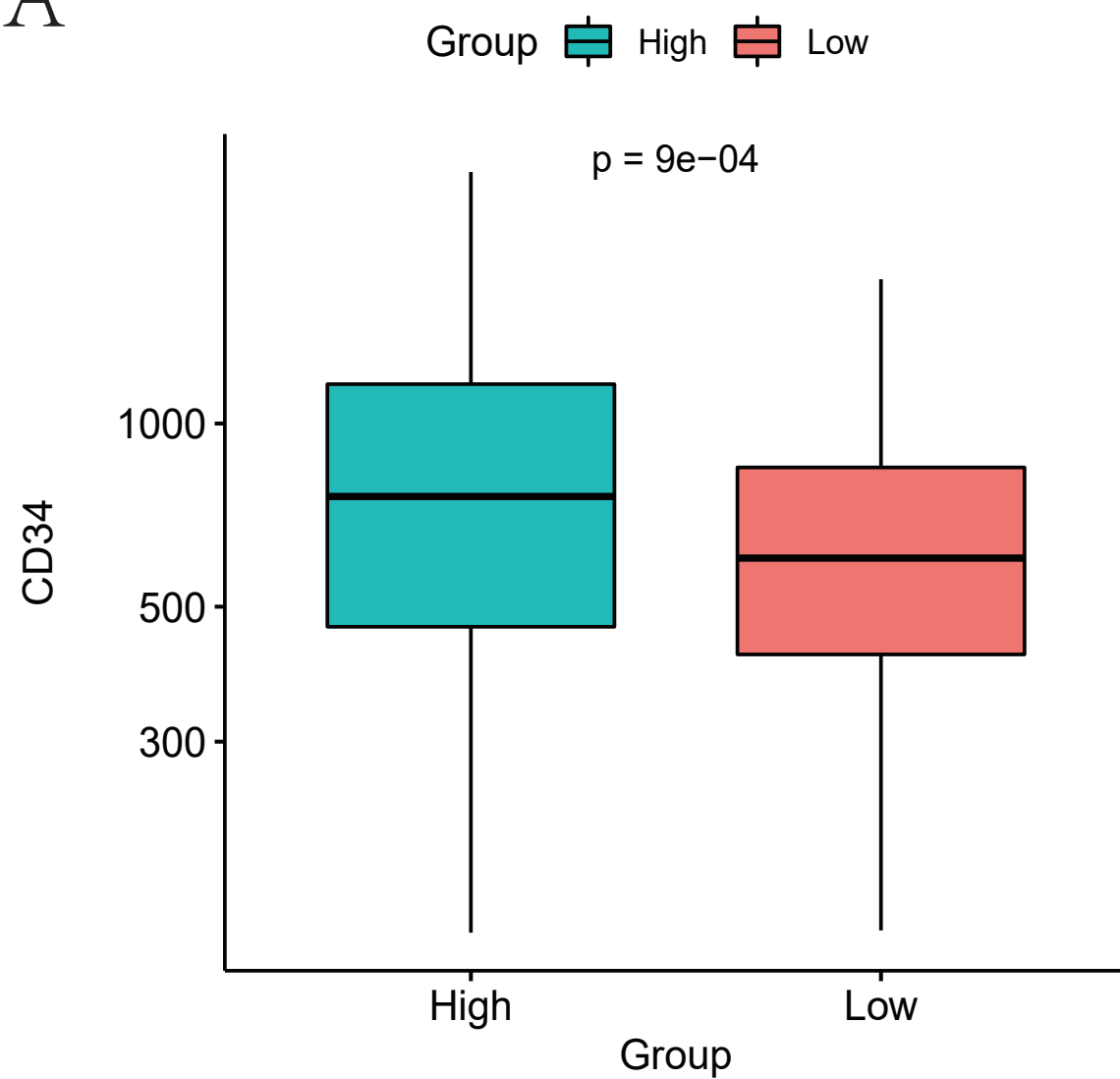

B

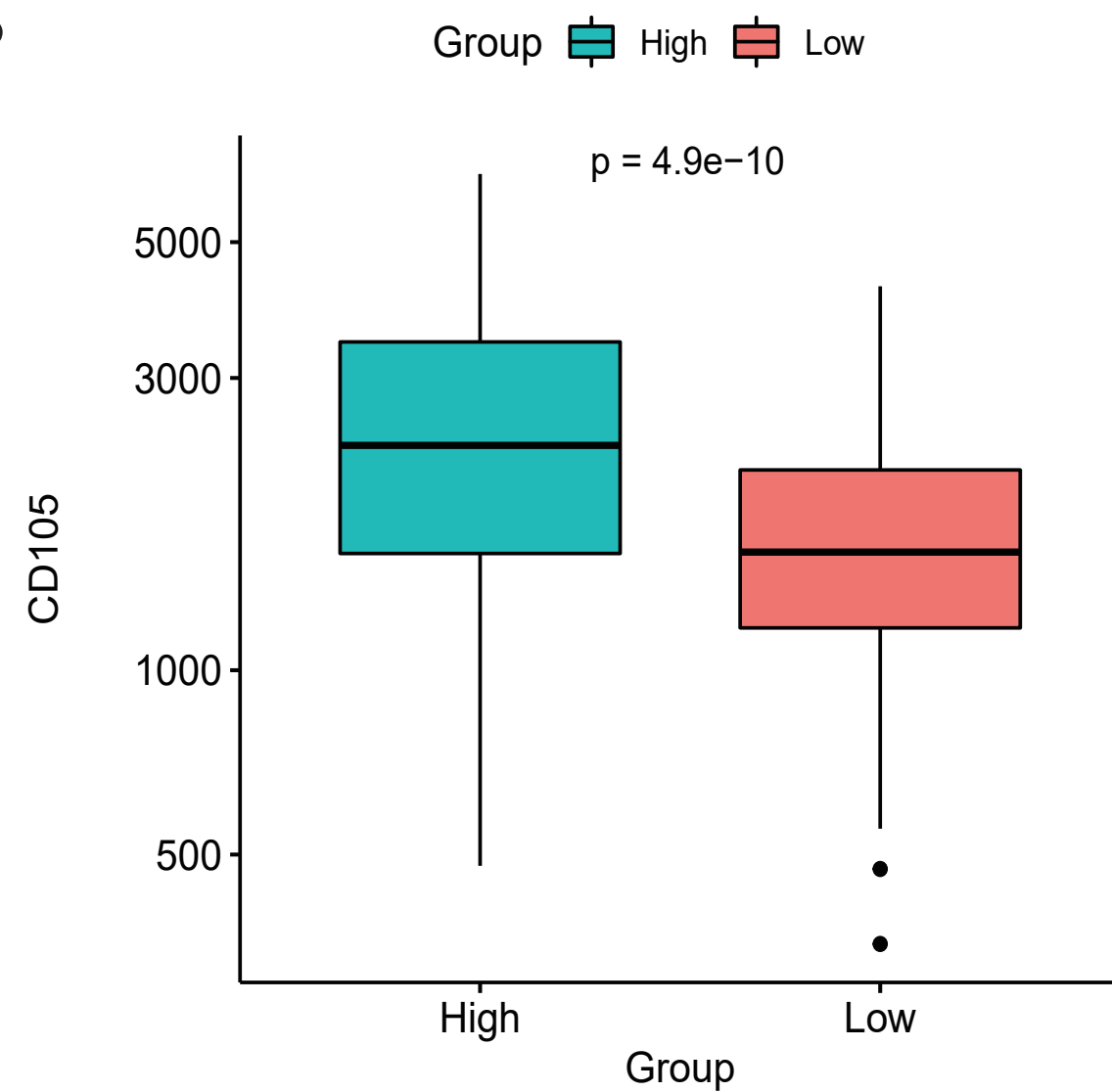

C

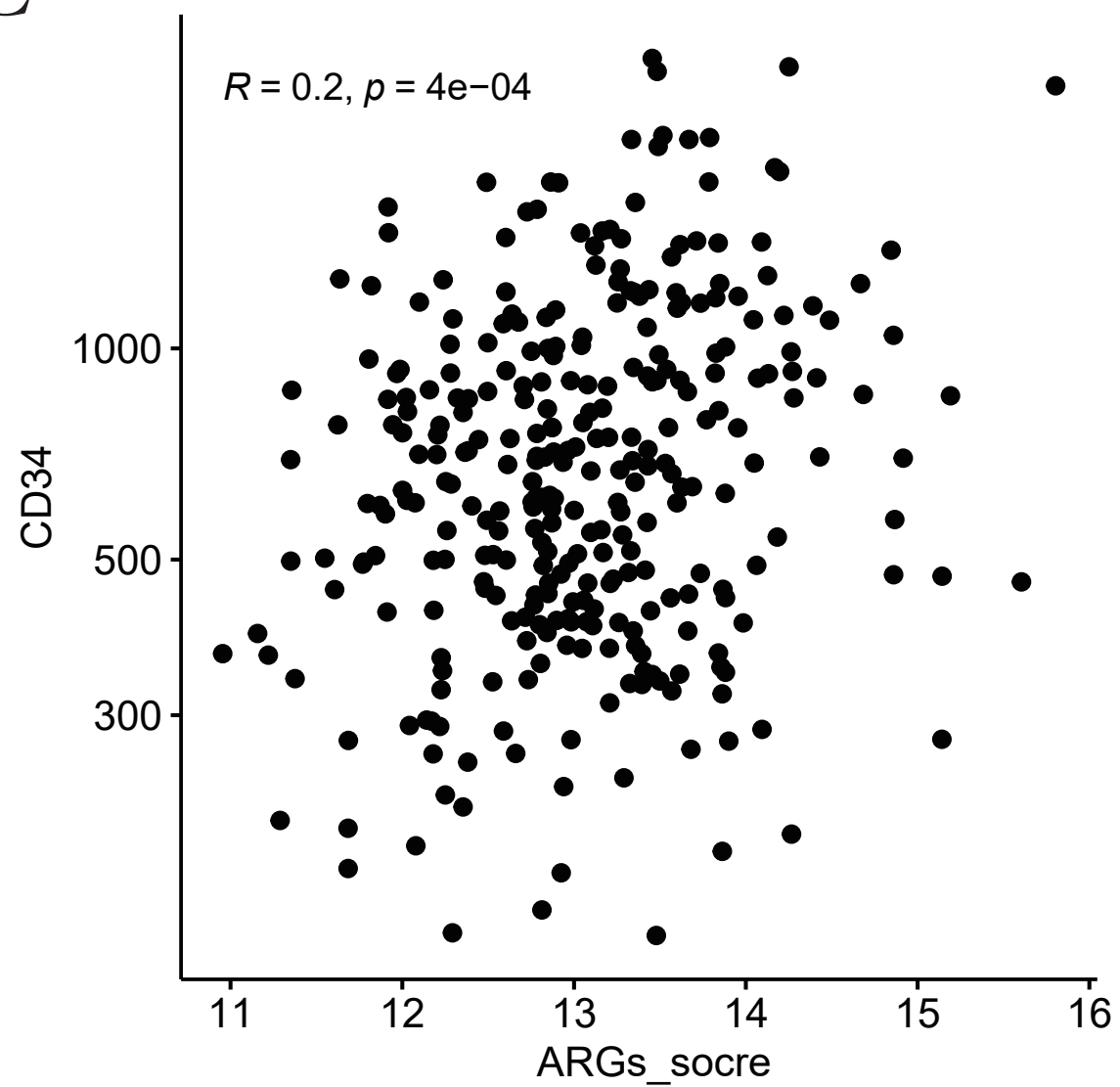

D

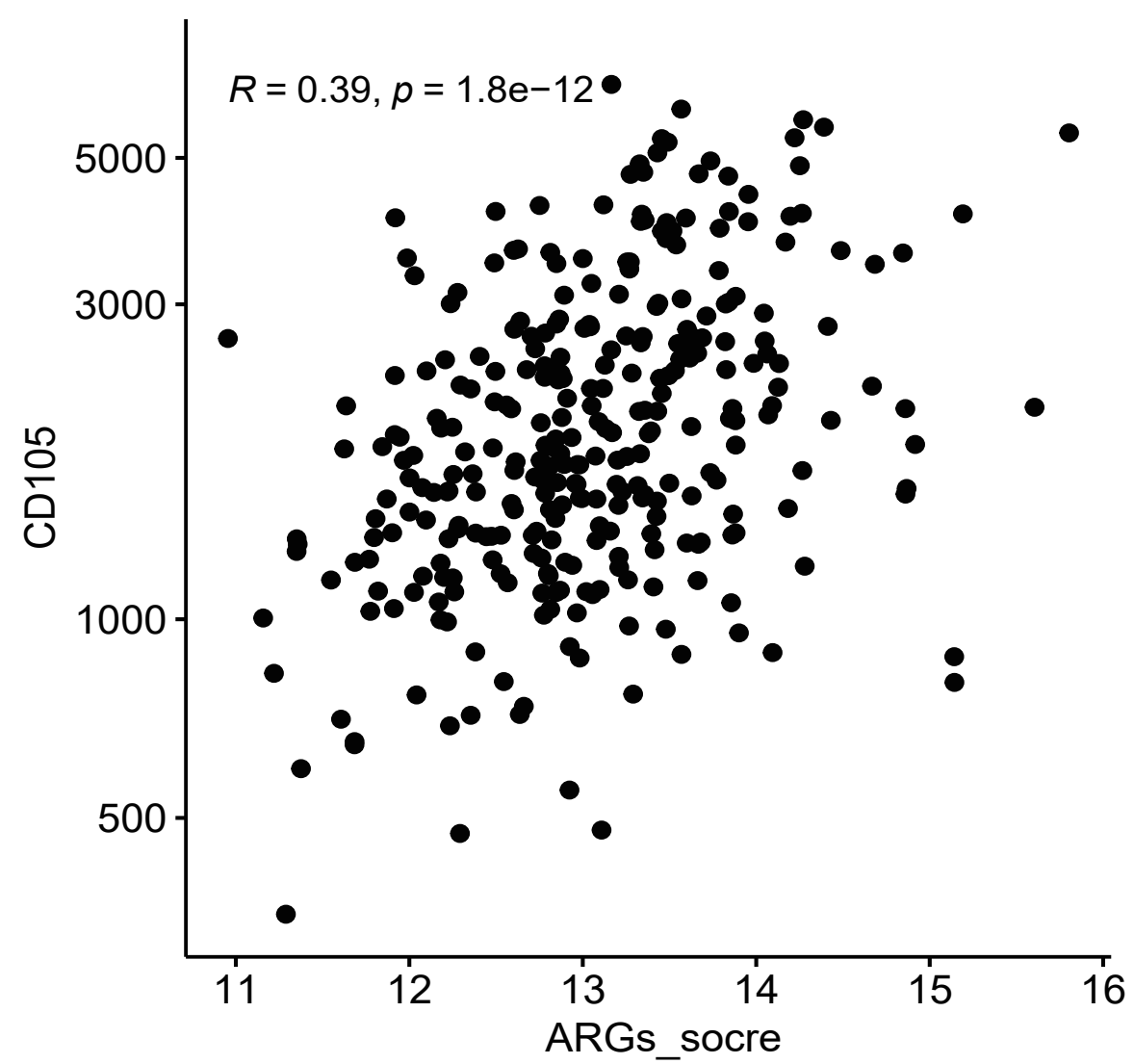

Supplement: Supplementary file 1 — Additional file 1. Fig. S1. The association between ARGs signature score and the expression of common tumor angiogenesis markers of CD34 & CD105. [file 10020_2022_504_MOESM1_ESM.pdf]

ROC curves for DFS at 1 year

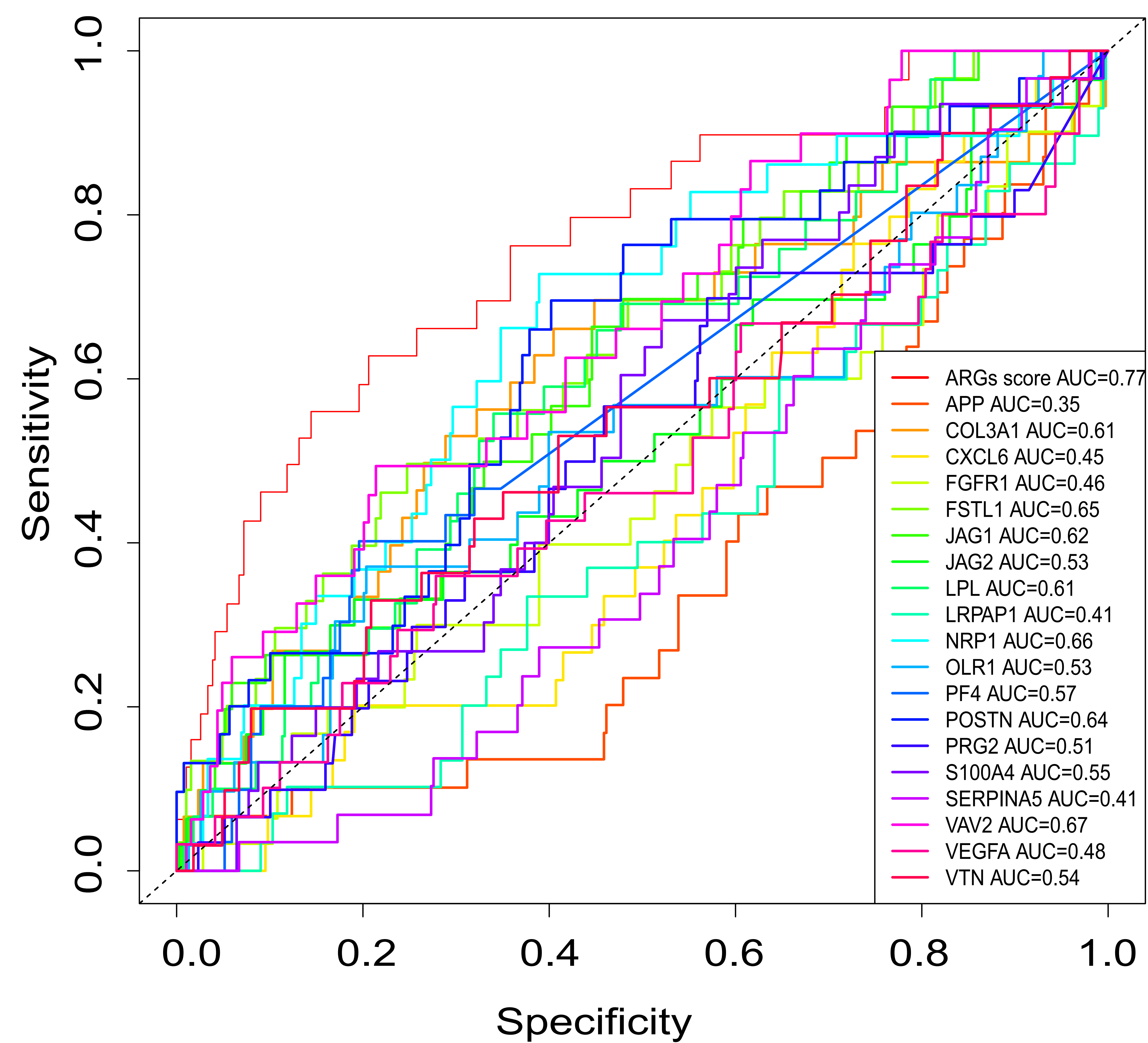

ROC curves for DFS at 3 years

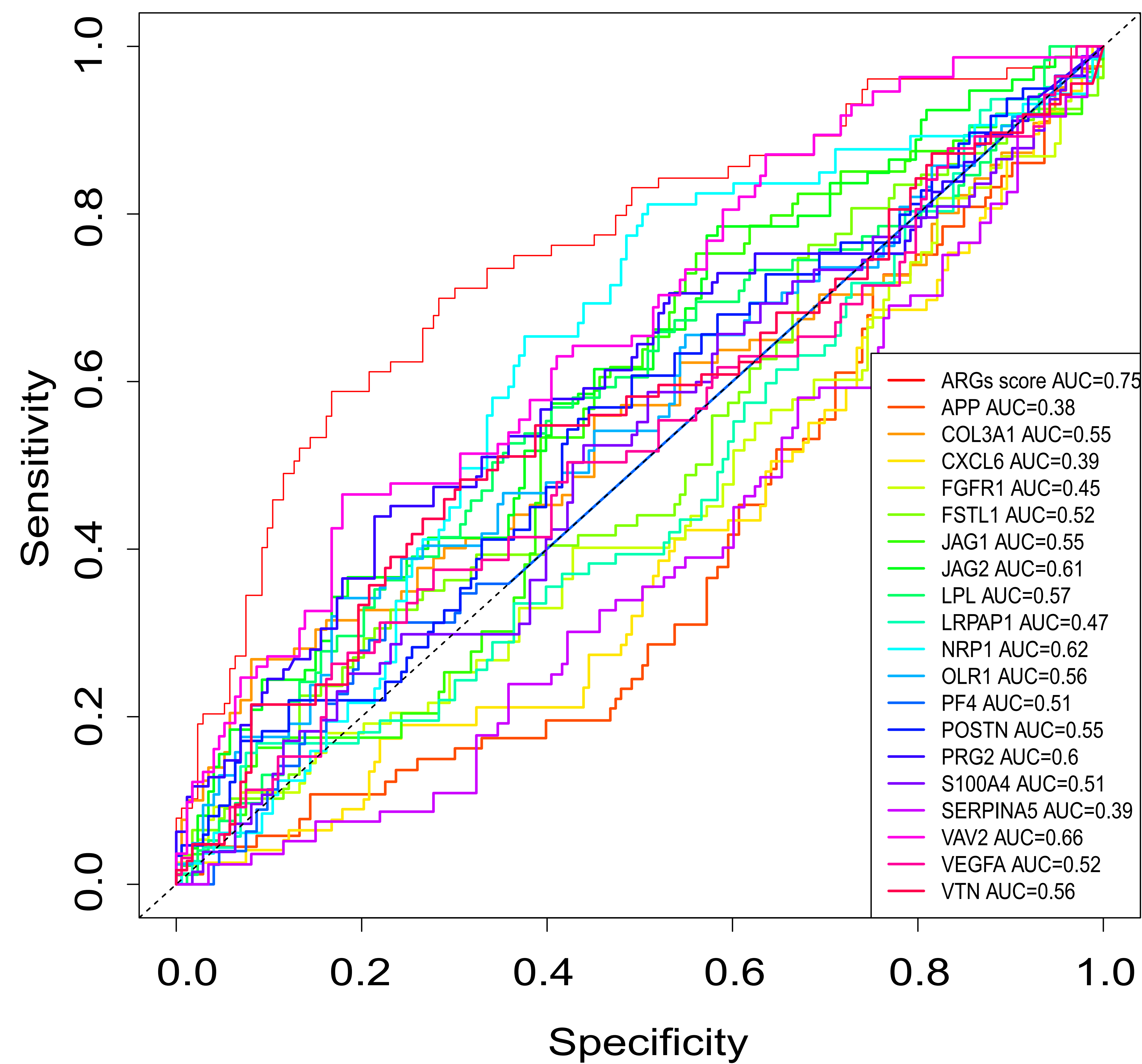

ROC curves for DFS at 5 years

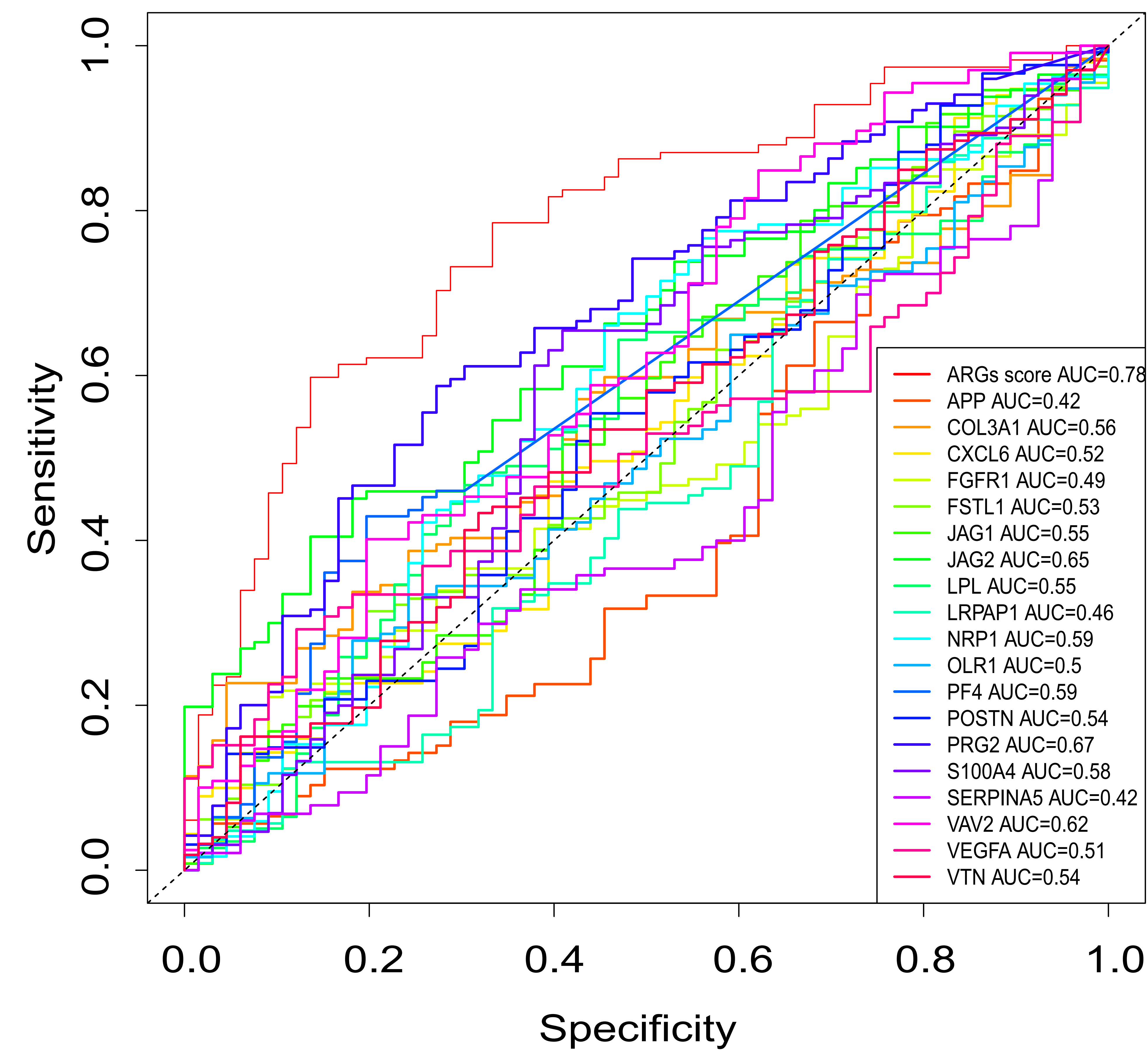

Supplement: Supplementary file 2 — Additional file 2. Fig. S2. The comparison between ARGs signature and each ARG in prognosis prediction via the receiver operating characteristic curve (ROC) analysis. [file 10020_2022_504_MOESM2_ESM.pdf]

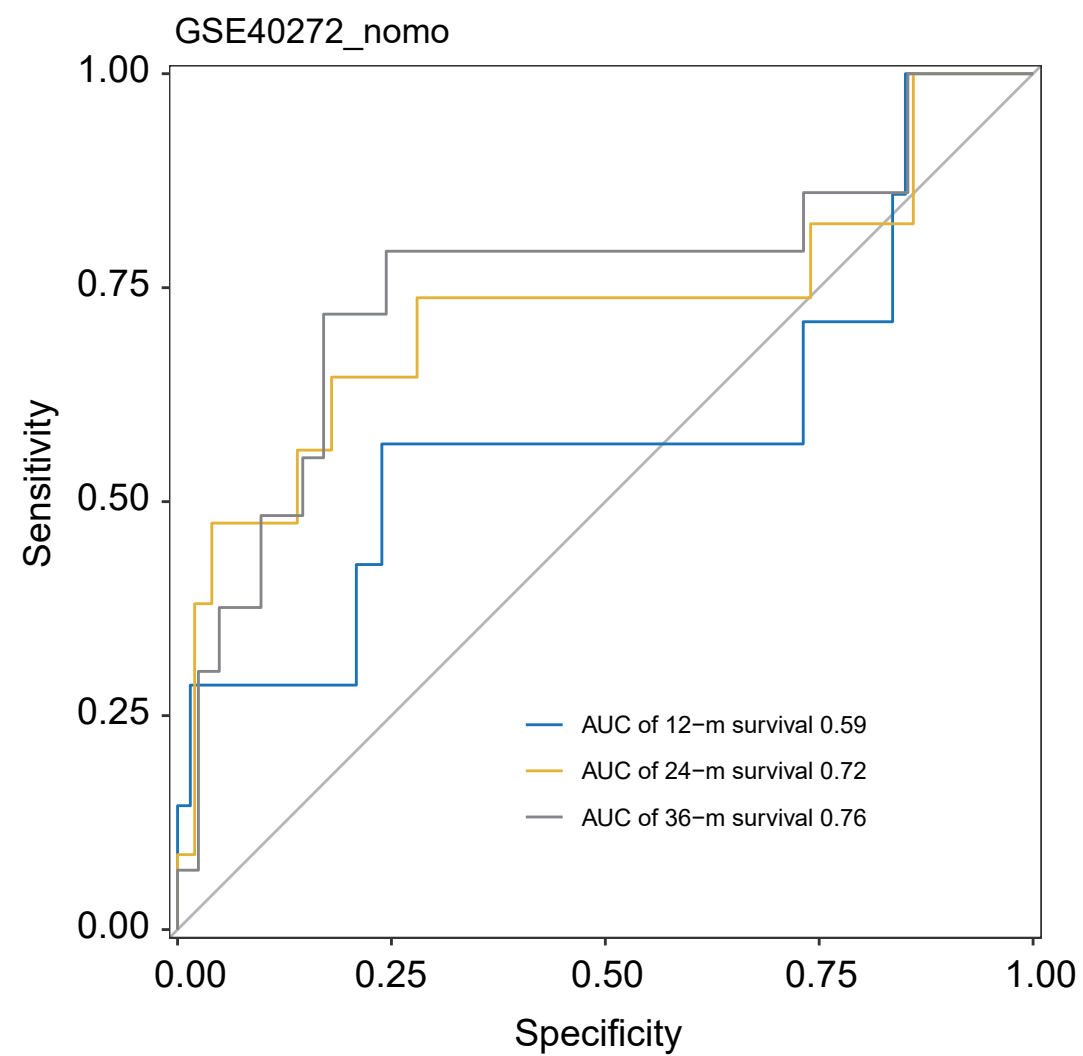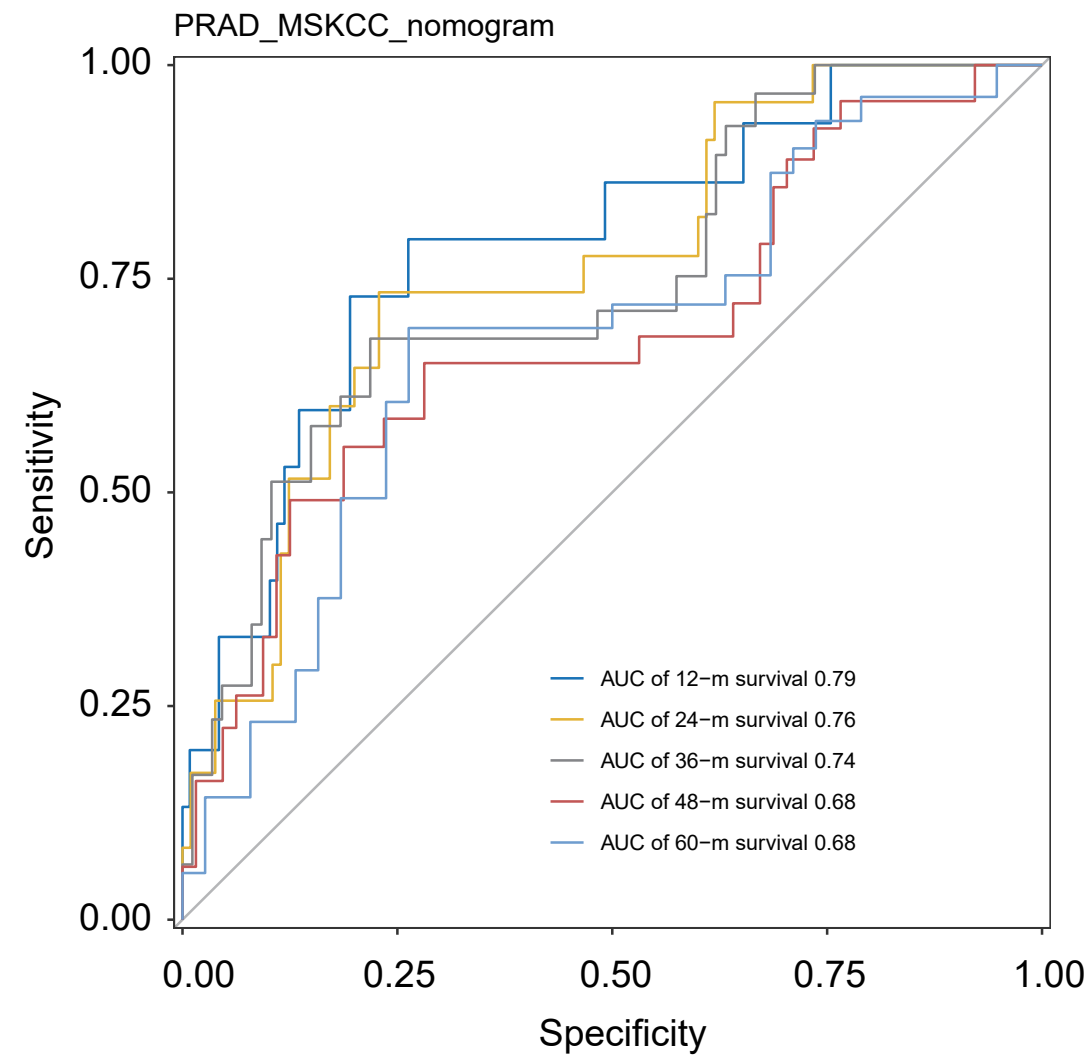

Supplement: Supplementary file 3 — Additional file 3. Fig. S3. The evaluation of nomogram in disease-free survival (DFS) prediction the receiver operating characteristic curve (ROC) analysis in GSE40272 and PRAD_MSKCC. [file 10020_2022_504_MOESM3_ESM.pdf]

Androgen Therapy   LHRH agonist   Orchiectomy   Untreated

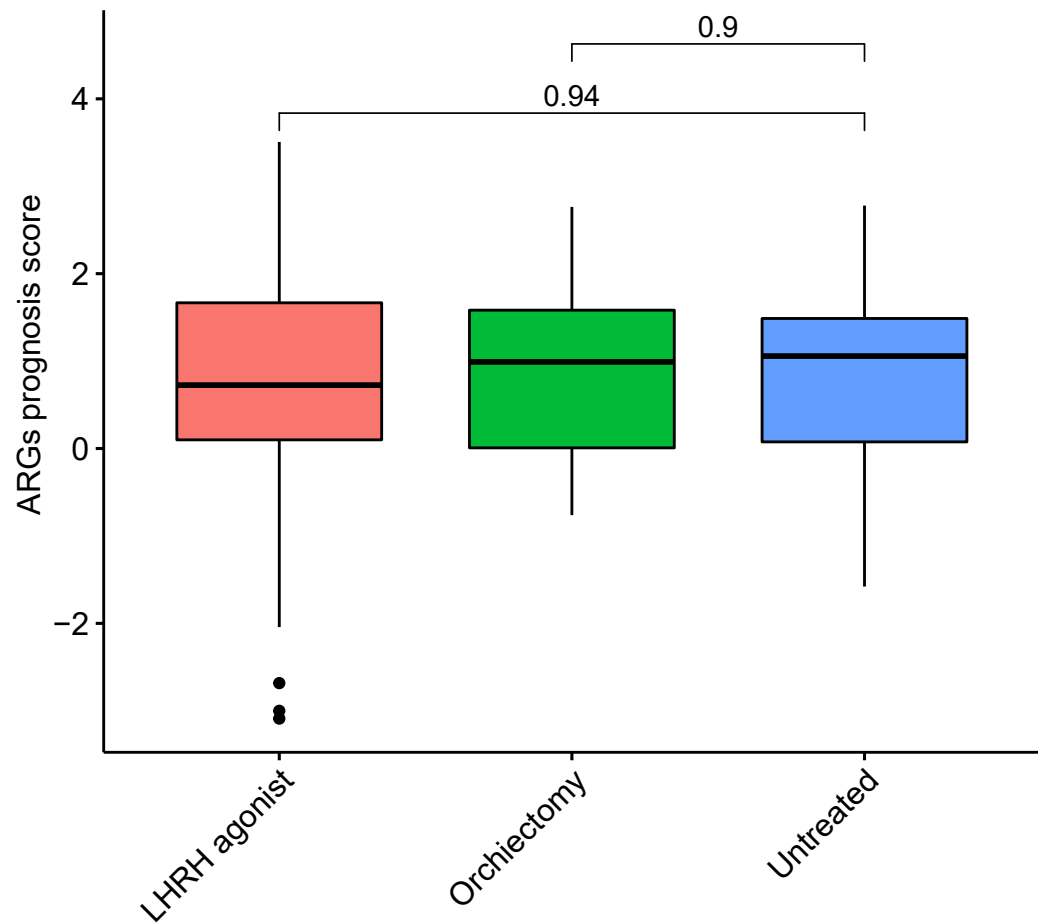

Supplement: Supplementary file 4 — Additional file 4. Fig. S4. The comparison of ARGs signature score between castration-resistant prostate cancer (CRPC) patients receiving luteinizing hormone-releasing hormone (LHRH) agonist or orchiectomy treatments, and some not receiving any therapies. [file 10020_2022_504_MOESM4_ESM.pdf]

PC3:docetaxel

$p = 1.2e-05$

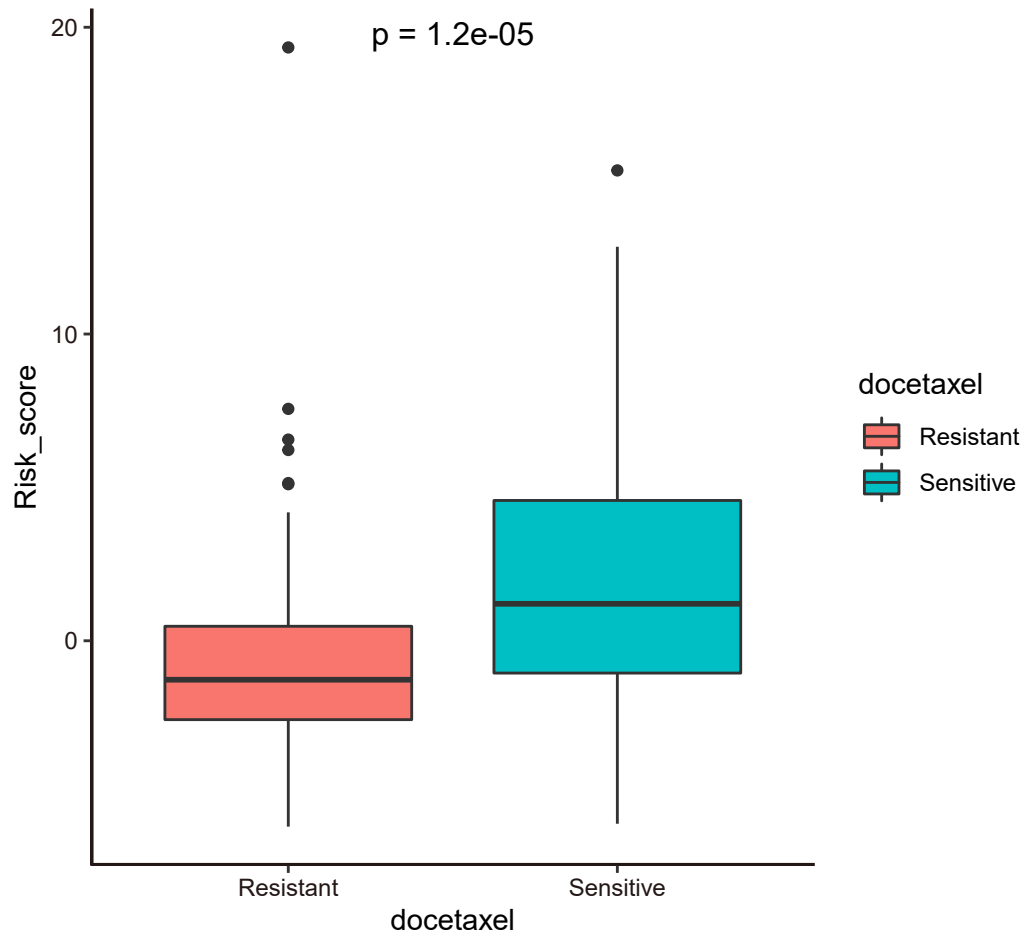

Supplement: Supplementary file 5 — Additional file 5. The validation of ARGs signature correlated with the sensitivity to docetaxel in PC3 PCa cell line in GSE140440. [file 10020_2022_504_MOESM5_ESM.pdf]

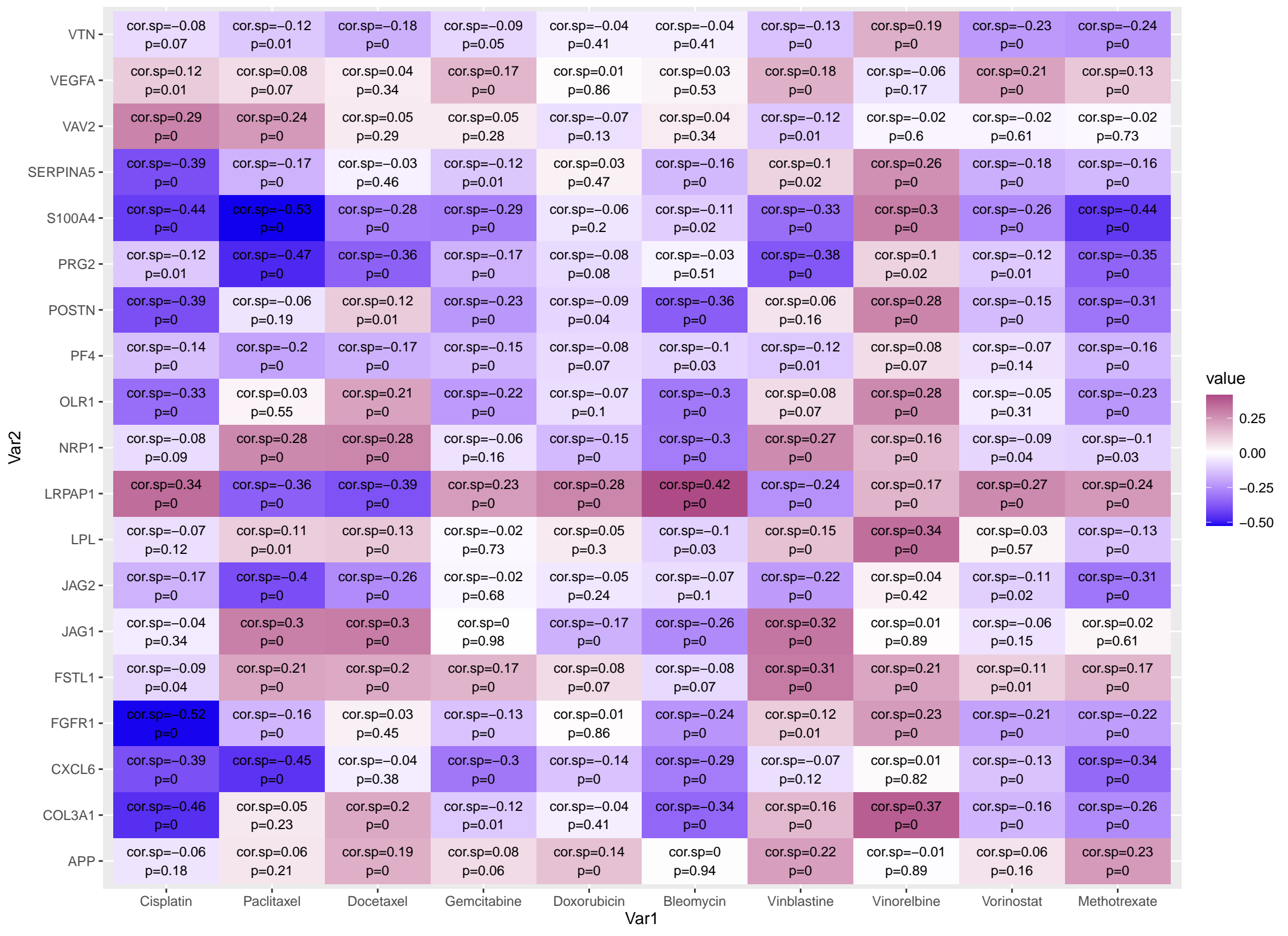

Supplement: Supplementary file 6 — Additional file 6. Fig. S6. The correlation analysis between each ARG expression and sensitivity to chemotherapy drugs, including cisplatin, paclitaxel, docetaxel, gemcitabine, doxorubicin, bleomycin, vinblastine, vinorelbine, vorinostat, methotrexate. [file 10020_2022_504_MOESM6_ESM.pdf]
