# Supplementary material for: The novel transcriptomic signature of angiogenesis predicts clinical outcome, tumor microenvironment and treatment response for prostate adenocarcinoma
Source: Mol Med. 2022 Jul 14;28:78. doi: 10.1186/s10020-022-00504-6 (PMC9284787; doi:10.1186/s10020-022-00504-6)
Supplement: Supplementary file 7 — Additional file 7: Table S1. The differentiation analysis in clinical characteristics of PRAD samples between the TCGA-PRAD cohort and GSE40272 dataset. [file 10020_2022_504_MOESM7_ESM.docx]

**Table S1.** The differentiation analysis in clinical characteristics of PRAD samples between the TCGA-PRAD cohort and GSE40272 dataset

| **Characteristic** |  | **TCGA** | **GSE40272** | **P-value** |
| --- | --- | --- | --- | --- |
| Total |  | 497 | 89 |  |
| Age | Median (range) | 61 [41, 78] | 62 [43, 73] | 0.9104 |
| PSA | Median (range) | 0.1 [0, 323] | 5.1 [2.1, 44.5] | <0.0001 |
| Gleason score | Primary + Secondary |  |  | <0.0001 |
|  | 6 | 45 | 13 |  |
|  | 7 | 247 | 62 |  |
|  | 8 | 64 | 4 |  |
|  | 9 | 137 | 6 |  |
|  | 10 | 4 | 0 |  |
| T stage | T1 | 177 | 0 | <0.0001 |
|  | T2 | 173 | 64 |  |
|  | T3 | 53 | 19 |  |
|  | T4 | 2 | 1 |  |
| N stage | N0 | 345 | 79 | 0.0034 |
|  | N1 | 79 | 5 |  |
| M stage | M0 | 455 | 84 | 0.1792 |
|  | M1 | 3 | 2 |  |

PRAD: prostate adenocarcinoma.
